# Supplementary material for: Including autapomorphies is important for paleontological tip-dating with clocklike data, but not with non-clock data
Source: PeerJ. 2018 Apr 6;6:e4553. doi: 10.7717/peerj.4553 (PMC5890724; doi:10.7717/peerj.4553)
Supplement: Supplemental Information 1 [file peerj-06-4553-s001.pdf]

Supplemental Material for: "Including autapomorphies is important for tip-dating with clocklike data, but not with non-clock data", by Nicholas J. Matzke and Randall B. Irmis

Supplemental Methods

The primary purpose of this study was not to conduct as rigorous a divergence dating study as possible; rather, it was to study the effects of inclusion/exclusion of autapomorphies and ascertainment bias corrections on tip-dating analyses. We suspect that for the present dataset, the autapomorphies issue is less problematic for dating than unresolved issues surrounding use of higher taxa as OTUs in a Birth-Death-Serial Sampling (BDSS) dating analyses, as well as the fact that for the Müller and Reisz dataset, taxa are sampled very non-uniformly in time.

Morphological matrix. Müller and Reisz (2006) assembled a matrix of 25 taxa (eureptiles and outgroups) and 132 characters, using personal and literature observations. Of these characters, 90 were parsimony-informative, and 42 were autapomorphic. We downloaded the matrix from TreeBase (Piel et al. 2002) (www.treebase.org; study accession no. S1462, matrix accession no. M2628) and converted it to simplified NEXUS and TNT formats. The same was done for a subset of the matrix that excluded autapomorphies. Summary statistics (e.g. completeness) were also calculated. All data manipulation was performed using *BEASTmasteR* functions (Matzke 2015a) and custom R scripts (Supplemental Data).

Tip dates. The focal issue of use of autapomorphies would remain unaffected even if substantial effort were put into resolving difficult issues surrounding the interpretation of OTUs and OTU dates in tip-dating analyses. However, taking the OTUs in the Müller and Reisz (2006) (Müller & Reisz 2006) data matrix as fixed, we assigned age ranges to the best of our ability, fully justifying them using peer-reviewed literature and the "best practices" guidelines of Parham et al. (2012) (2012). Ages, justifications, and supporting references are provided in Supplemental Table S1, and in Supplement Data as an Excel spreadsheet.

In addition, a preliminary version of this analysis was conducted using databased stratigraphic ranges. Here, date ranges for each taxon were gathered from Fossilworks/PaleobioDB (http://www.fossilworks.org/ and http://paleodb.org/; accessed December 24, 2015; (Alroy 2016; Alroy et al. 2016)). The exception was Seymouriamorpha, which could not be found in the database; therefore the date range for *Seymouria* was used for this tip. Stratigraphic ranges were interpreted as Uniform(min, max) priors on each tip age. We prefer the analysis based on "best practices" ages discussed above, and emphasize this analysis in the main text, but include the ages used in the preliminary analysis in Supplemental Table 2 and Supplemental Data, and the results below.

Note on tip-dating analyses when no OTUs reach the Recent. The coherence of the date inputs in a tip-dating study depends on an absolute time reference. Normally, this role is played by extant taxa with a date of 0 Ma; however, as this dataset consists entirely of fossils, the youngest tip in the dataset, Procolophonidae, was given a fixed date at the youngest edge of its stratigraphic range (201.6 Ma, the age of the end-Triassic mass extinction when procolophonids

went extinct. In the actual *Beast2* XML file, 201.6 Ma was subtracted from all dates, making Procolophonidae age 0 in the BEAST analysis. This age was added back to all dates post-analysis. This procedure has the disadvantage of treating the age of one of the OTUs as fixed, but we are unsure how Beast2 deals with the issue of relative versus absolute dates in the situation where all tips have uncertain dates, and in any case, valid interpretation and comparison of the output Newick strings absolutely requires an absolute time reference.

Maximum Parsimony (MP) analysis. Cladistic analyses were run on the complete and parsimony-informative datasets in TNT (Goloboff et al. 2008) using scripts similar to those used for the parsimony analysis in Matzke (2016) (Matzke 2015b; Matzke 2016). Search settings were determined by a cosmetically-altered version of the *aquickie.run* script included in the TNT download. The tree search was run with *xmult* plus 10 cycles of tree drifting (Goloboff 1999) with 20 independent searches for the optimal score. Both datasets were run without using the *xinact* command, so that any differences between the two datasets (for example, in terms of branchlengths in number of steps) could be observed. The script also calculated Bremer and bootstrap supports on the strict consensus topology, and additions to the script used TNT commands to calculate morphological branchlengths, plotted synapomorphies, calculated homoplasy, Consistency Index (CI), and Retention Index (RI) on the entire datasets and by character. All results were output to a plain-text logfile, which was processed by TNTR functions (Matzke 2015b) in a custom R script (Supplemental Data) to produce annotated Newick files, summary tables as tab-delimited text files, and PDF graphics.

Test for correlation between time and character change. One assumption of tip-dating analyses is that the degree of morphological change will correlate to some extent with geologic time. A rough check on this is to regress the age of each tip (taking the expected mean age of the OTU, if a distribution was specified) against the number of steps above the root for each tip in the MP analysis. This was performed on the reduced and full datasets. We do not mean to put too much weight on this regression test, as both tip age, and number of steps above the root, are likely to exhibit phylogenetic autocorrelation. However, the simple regression can help serve at least as a check to see to what degree tips higher in the stratigraphic record tended to have accumulated more evolutionary changes, in a particular character matrix. Methods for correcting for phylogenetic autocorrelation might be attempted in the future. Unfortunately, simple methods like phylogenetic independent contrasts (Felsenstein 1985) are designed for continuous characters evolving under Brownian motion assumptions, and are not directly applicable to this problem, although they might inspire solutions.

Priors and settings for the Beast2 run. The Birth-Death Serial Sampling (BDSS) tree prior was used for all analyses; BDSS-SA (adding sampled ancestors; (Gavryushkina et al. 2015; Gavryushkina et al. 2014)) was not used, as the taxonomic spread and small number of OTUs in the dataset makes it unlikely that any OTU is a direct ancestor of another. Priors on birth, death, sampling, and clock rates were set to Uniform(0,10). A relaxed morphological clock with lognormally-distributed branch rate variation was used for all analyses. All priors and model decisions for each run, including the date ranges for OTUs, are available in the *BEASTmasteR* settings Excel file (Supplemental Data). *BEASTmasteR* and its precursors have been used to

generate XML tip-dating analyses in a series of publications (Alexandrou et al. 2013; Close et al. 2015; Guillerme & Cooper 2016; Puttick et al. 2016; Sánchez et al. 2015; Wood et al. 2013).

Starting tree for Beast2 analyses. Obtaining starting trees that would match all of the "hard" constraints mandated by the uniform tip-date priors proved difficult, so initial analyses were conducted using a random starting tree, with all of the uniform date constraints converted to "soft" normal distributions. This was effected by setting the "convert_to_normal" field to "yes" for each tip-date in the *BEASTmasteR* Excel settings file, an option created to avoid tedious manual re-setting of the prior for each tip-date in *BEAUTi*. Once this analysis had burned in, the last sampled tree served as a valid starting tree under the hard constraints.

Implementation of M*kv and* M$k_{parsinf}$*.* We implemented M*k*v and Mk_{parsinf} in *BEASTmasteR* with functions (*num_unobservable_patterns_ParsInf*, *list_unobservable_patterns_ParsInf*, *make_table_num_unobservable_patterns*) that list all unobservable site patterns under these corrections as dummy characters. The dummy characters are included in the *Beast2* XML using the "ascertained," "excludefrom," and "excludeto" options of the "data" XML tag.

MCMC runs. The relatively small size of the morphological dataset and the large uncertainties in some tip-dates necessitated substantial sampling to achieve burn-in and sufficient mixing. However, the small size of the data also sped calculation, so each analysis was run for 10^9 generations, with each run taking approximately 12 hours on a 2015 Mac Pro, for the M*k* and M*k*v analyses. However, the Mk_{parsinf} ascertainment bias correction requires calculating the likelihood over all unobservable site patterns. For the Müller and Reisz (2006) dataset (Müller & Reisz 2006), this adds up to 52 additional site patterns for the 2-state characters, and 1953 additional patterns for the 3-state characters (calculated with the function *num_unobservable_patterns_ParsInf*; see Appendix 1). An Mk_{parsinf} run of 10^9 generations took approximately 15.25 days on the same machine.

Trees and parameters were sampled every 500,000 generations, resulting in 2000 saved samples. Burn-in was achieved well before this, but to be conservative, the first 25% of the samples were discarded for calculating the summary tree and parameter 95% highest posterior densities (HPDs).

Simulation analysis. In order to test our explanation of why excluding autapomorphies did not influence dating inference in the empirical eureptiles dataset, we constructed two simulation that would dramatically show the difference between a clocklike and non-clocklike dataset.

The dated phylogeny, including fossil tips, was simulated under a BDSS-skyline model with *TreeSim*, using these settings:

```
numtips=30
lambdasky = c(0.6, 0.6, 0.3)
deathsky = c(0.3, 0.3, 0.15)
sampprobsky = c(2, 2, 0.01)
```

```
timesky = c(10, 20, 30)

seedval = 543210
set.seed(seed=seedval)
tr = sim.bdsky.stt(n=numtips, lambdasky, deathsky=deathsky,
timesky=timesky, sampprobsky=sampprobsky,
omegasky=rep(0,length(timesky)), rho=0, timestop=0, model="BD",
N=0, trackinfecteds=FALSE)
```

A tree size of 30 species was chosen to roughly mirror the Müller/Reisz dataset. Sampling was also focused earlier in the tree, to somewhat resemble the situation observed in the Müller/Reisz dataset (this was not particularly successful; the distribution of sampled tips in that dataset is very far from what BDSS simulations will produce, due to the preponderance of tip-dates in a narrow time range).

Two character datasets were simulated on the phylogeny, using *sim.char* from the R package *ape*:

1. "strict clock" -- simulate 1000 binary characters under an M*k* model. The rate was set to be low enough (0.05) that many characters (577/1000) are invariant.

2. "non-clock" -- the time branchlengths on the true tree were randomly reshuffled once. The 1000 characters were then simulated on this new tree (by chance, again 577/1000 were invariant). The tip dates are still from the true tree.

Filtering the character datasets according to the possible ascertainment biases produced:

- two "alldata" datasets of 1000 characters of all types
- two "variable" datasets of 423 variable characters (both had 423, by chance)
- two "informative" datasets of 190 (strict clock) and 352 (no clock) characters

Twelve *Beast2* inference runs (BDSS, lognormal relaxed clock, gamma-distributed sitewise rate variation with 4 sites – the same settings as for the empirical eureptile analyss) were then done, using each applicable combination of Mk, Mkv, and MkParsInf on each of these datasets.

The names of the folders in Supplemental Files containing these analyses are as follows.

On the strict clock dataset:

01_Mk_on_strict_clock_alldata
02_Mk_on_strict_clock_variable
03_Mk_on_strict_clock_informative
04_Mkv_on_strict_clock_variable
05_Mkv_on_strict_clock_informative

06_MkParsInf_on_strict_clock_informative

On the non-clock dataset:

07_Mk_on_no_clock_alldata
08_Mk_on_no_clock_variable
09_Mk_on_no_clock_informative
10_Mkv_on_no_clock_variable
11_Mkv_on_no_clock_informative
12_MkParsInf_on_no_clock_informative

Each run took 100 million generations, sampling every 50,000. The trace files were inspected for convergence in Tracer (all converged easily), MCC trees were generated with TreeAnnotator, and the results were compared with custom R scripts (Supplemental Files; directory _06_TreeSim).

Supplemental Results

Morphological matrix. The data matrix was 84.64% complete, or 82.76% complete when autapomorphies were excluded (Supplemental Table S3).

Parsimony analysis. As expected, parsimony analyses of the two datasets (Figs S1-S10) yielded an identical strict consensus tree, with bootstrap values very similar to that of Müller and Reisz (2006) (Müller & Reisz 2006). They reported finding 4 trees with length (TL) of 252 parsimony-informative steps, CI=0.4405, and RI=0.6493. Here, the TNT analysis yielded found 2 trees with TL=253, CI=0.395, and RI=0.619. This may be due to a slight difference in the TreeBase dataset, or calculating the statistics on the 50% majority rule consensus tree (Müller & Reisz) versus the strict consensus tree (this study), which is slightly less resolved. As expected, MP trees had identical topology between the full and no-autapomorphies datasets, but some terminal branches were longer in the full dataset (compare Fig. S1 vs. S6; the whole tree was also longer: TL=295, i.e. 42 steps longer, corresponding to adding 42 autapomorphies). The most dramatic example of this was Procolophonidae, for which the terminal branchlength changed from 9 to 22 steps (Fig. S6).

Test for correlation between time and character change. Figures S11 and S12 show the results of regression of tip age on the number of parsimony character steps above the root. Neither dataset shows impressive correlation with time, probably in part because of the uncertainty in some OTU time ranges, and because many of the sampled taxa come from about the same time period, 315-300 Ma. Nonetheless, including autapomorphies does make the relationship somewhat more positive (without autapomorphies: slope=0.08, R^2=0.015, P=0.56; with autapomorphies: slope=0.17, R^2=0.067, P=0.21).

If the main purpose of this study were a “best possible” dating effort, the observed lack of correlation would be a reason for concern (although, in tip-dating, a substantial part of the signal informing the dating inference is from the dates of the tips, not only the morphological clock model), along with the non-uniform sampling of OTUs in time. However, the primary goal of the present study is to compare the impact of autapomorphy-related decisions on otherwise identical datasets.

Changes in estimates of morphological relaxed clock model parameters. Although the difference in clock rates between autapomorphy-including and autapomorphy-excluding runs is sufficiently obvious that no test is needed (the average clock rate estimate is 10 times higher when autapomorphies are excluded), some readers may prefer that the tests be presented. Tests were run on post-burnin MCMC samples of the clock mean rate parameter (N=1500 post-burnin samples of clock rate, which are uncorrelated in this case, due to only 1500 samples being taken from a run of 10^9). Testing the null hypothesis of no difference in means between the samples of clock rates yielded P<2.2e-16 for all variants, using *t*-tests and Wilcoxon signed rank tests, on both untransformed and log-transformed rate samples. All tests were two-sided and non-paired. The standard deviation of the relaxed clock rate across branches is also higher in the no-autapomorphies dataset (Table 1, all P<2.2e-16, same tests).

Comparison of dates and HPDs across all 10 runs. On this dataset, results using PaleobioDB dates closely paralleled those using best-practices dates (e.g., Fig. 1e), although dating uncertainty was higher for some tips and nodes. Figure S13 contains the pairwise correlation plots between the mean node ages for the shared nodes found in the summary trees of all 10 analyses (5 analyses under the best-practices tip dates, plus the same 5 analyses under the PaleobioDB tip dates). Figure S14 contains the same for the 95% HPD widths.

Supplemental Discussion

The most dramatic difference in parameter inference on the eureptile dataset was the order-of-magnitude difference in the mean rate parameter of the relaxed morphological clock model between analyses that include, or exclude, autapomorphies. The reason for this difference seems clear. In morphological clock analyses, autapomorphic characters are interpreted as low-rate characters. In parsimony terms, each autapomorphic state only occurred once and thus comprises only one change on the tree. In probabilistic models, there is a chance for multiple changes, but the expectation is nevertheless low. Some parsimony-informative characters, on the other hand, might have the minimum number of changes, if they exhibit no homoplasy. However, in real datasets, homoplasy is common, and this will translate to higher rates in a model-based framework. The inclusion of 42 low-rate characters in the data matrix thus shifts the distribution of rates downward, as well as the overall mean.

The effect of this change in rates on inferred dates, date uncertainty, and posterior probabilities were modest in the studied dataset. However, they are predictable consequences of the models employed in tip-dating, and thus important to understand. The increase in clock rate

under a no-autapomorphies dataset detectably decreased bipartition posterior probabilities, and increased dating uncertainty. The increase in rates has these effects because increasing rates increases the probability of multiple convergent character changes, and thus increases the probability of alternate topologies that fit the character data almost equally well. This translates into increased uncertainty.

Supplemental References

Alexandrou MA, Swartz BA, Matzke NJ, and Oakley TH. 2013. Genome duplication and multiple evolutionary origins of complex migratory behavior in Salmonidae. *Molecular Phylogenetics and Evolution* 69:514-523. 10.1016/j.ympev.2013.07.026

Alroy J. 2016. Fossilworks. Gateway to the Paleobiology Database. http://www.fossilworks.org/

Alroy J, Marshall C, and Miller A. 2016. Paleobiology Database. Produced by NCEAS. https://paleobiodb.org

Close Roger A, Friedman M, Lloyd Graeme T, and Benson Roger BJ. 2015. Evidence for a mid-Jurassic adaptive radiation in mammals. *Current Biology* 25:2137-2142. 10.1016/j.cub.2015.06.047

Felsenstein J. 1985. Phylogenies and the comparative method. *The American Naturalist* 125:1-15.

Gavryushkina A, Heath TA, Ksepka DT, Stadler T, Welch D, and Drummond AJ. 2015. Bayesian total evidence dating reveals the recent crown radiation of penguins. *arXiv*. arXiv:1506.04797

Gavryushkina A, Welch D, Stadler T, and Drummond AJ. 2014. Bayesian inference of sampled ancestor trees for epidemiology and fossil calibration. *PLoS Comput Biol* 10:e1003919. 10.1371/journal.pcbi.1003919

Goloboff PA. 1999. Analyzing large data sets in reasonable times: solutions for composite optima. *Cladistics* 15:415-428.

Goloboff PA, Farris JS, and Nixon KC. 2008. TNT, a free program for phylogenetic analysis. *Cladistics* 24:774-786. 10.1111/j.1096-0031.2008.00217.x

Guillerme T, and Cooper N. 2016. Effects of missing data on topological inference using a total evidence approach. *Molecular Phylogenetics and Evolution* 94, Part A:146-158. 10.1016/j.ympev.2015.08.023

Matzke NJ. 2015a BEASTmasteR: automated conversion of NEXUS data to BEAST2 XML format, for fossil tip-dating and other uses. PhyloWiki. http://phylo.wikidot.com/beastmaster

Matzke NJ. 2015b TNTR: R functions to aid analyses in the cladistics program TNT. PhyloWIki. http://phylo.wikidot.com/tntr

Matzke NJ. 2016. The evolution of antievolution policies after *Kitzmiller versus Dover*. *Science* 351:28-30. 10.1126/science.aad4057

Müller J, and Reisz RR. 2006. The phylogeny of early eureptiles: comparing parsimony and Bayesian approaches in the investigation of a basal fossil clade. *Systematic Biology* 55:503-511. 10.1080/10635150600755396

Parham JF, Donoghue PCJ, Bell CJ, Calway TD, Head JJ, Holroyd PA, Inoue JG, Irmis RB, Joyce WG, Ksepka DT, Patané JSL, Smith ND, Tarver JE, van Tuinen M, Yang Z, Angielczyk KD, Greenwood JM, Hipsley CA, Jacobs L, Makovicky PJ, Müller J, Smith KT, Theodor JM, Warnock RCM, and Benton MJ. 2012. Best practices for justifying fossil calibrations. *Systematic Biology* 61:346-359. 10.1093/sysbio/syr107

Piel WH, Donoghue MJ, and Sanderson MJ. 2002. TreeBASE: a database of phylogenetic knowledge. In: Shimura J, Wilson KL, and Gordon D, eds. *To the Interoperable "Catalog of Life" with Partners Species 2000 Asia Oceanea*, 41-47.

Puttick MN, Thomas GH, and Benton MJ. 2016. Dating placentalia: morphological clocks fail to close the molecular fossil gap. *Evolution*. 10.1111/evo.12907

Sánchez IM, Cantalapiedra JL, Ríos M, Quiralte V, and Morales J. 2015. Systematics and evolution of the Miocene three-horned palaeomerycid ruminants (Mammalia, Cetartiodactyla). *PLoS ONE* 10:e0143034. 10.1371/journal.pone.0143034

Wood HM, Matzke NJ, Gillespie RG, and Griswold CE. 2013. Treating fossils as terminal taxa in divergence time estimation reveals ancient vicariance patterns in the Palpimanoid spiders. *Systematic Biology* 62:264-284. 10.1093/sysbio/sys092

Supplemental Figure and Table Captions for: "Tip-dating studies should include autapomorphies"

Supplemental Figures

Supplemental Figure S1. Strict consensus tree from MP analysis of the autapomorphy-excluding dataset. Branchlengths, and the numbers on each branch, represent the number of parsimony steps as estimated by TNT.

Supplemental Figure S2. Strict consensus tree from MP analysis of the autapomorphy-excluding dataset. The numbers on each branch represent the number of synapomorphies as estimated by TNT.

Supplemental Figure S3. Strict consensus tree from MP analysis of the autapomorphy-excluding dataset. The numbers on each branch represent the Bremer support (decay index) as estimated by TNT's *aquickie.run* script.

Supplemental Figure 4. Strict consensus tree from MP analysis of the autapomorphy-excluding dataset. The numbers on each branch represent the relative Bremer support as estimated by TNT's *aquickie.run* script.

Supplemental Figure S5. Strict consensus tree from MP analysis of the autapomorphy-excluding dataset. The numbers on each branch represent the bootstrap support frequency (out of 100 trees) as estimated by TNT's *aquickie.run* script.

Supplemental Figure S6. Strict consensus tree from MP analysis of the autapomorphy-including dataset. Branchlengths, and the numbers on each branch, represent the number of parsimony steps as estimated by TNT.

Supplemental Figure S7. Strict consensus tree from MP analysis of the autapomorphy-including dataset. The numbers on each branch represent the number of synapomorphies as estimated by TNT.

Supplemental Figure S8. Strict consensus tree from MP analysis of the autapomorphy-including dataset. The numbers on each branch represent the Bremer support (decay index) as estimated by TNT's *aquickie.run* script.

Supplemental Figure S9. Strict consensus tree from MP analysis of the autapomorphy-including dataset. The numbers on each branch represent the relative Bremer support as estimated by TNT's *aquickie.run* script.

Supplemental Figure S10. Strict consensus tree from MP analysis of the autapomorphy-including dataset. The numbers on each branch represent the bootstrap support frequency (out of 100 trees) as estimated by TNT's *aquickie.run* script.

Supplemental Figure S11. Linear regression of tip height (mean of the prior distribution for that tip) against number of character steps above the root on the MP tree, for the autapomorphy-excluding dataset.

Supplemental Figure S12. Linear regression of tip height (mean of the prior distribution for that tip) against number of character steps above the root on the MP tree, for the autapomorphy-including dataset.

Supplemental Figure S13. Pairwise correlation plots between the mean node ages for the shared nodes found in the summary trees of all 10 analyses.

Supplemental Figure S14. Pairwise correlation plots between the node age HPD widths (95% highest posterior densities) for the shared nodes found in the summary trees of all 10 analyses.

Supplemental Figure S15. Comparison of the tip-dated phylogenies of early eureptiles inferred when excluding (a, b) or including (c, d) autapomorphies. (a, c): inference conducted under the M*k* model. (b, d): inference using M*k*v. Numbers are posterior probabilities. Bars represent the 95% HPD. (e): shows the result of $Mk_{parsinf}$ on the autapomorphies-excluded dataset, and (f) shows the same analysis using preliminary dates obtained from PaleobioDB, done in order to compare the effects of a "naïve" dating analysis with dates taken directly from a database, versus an expert review.

Supplemental Tables

Supplemental Table S1. Date ranges for Operational Taxonomic Units (OTUs) in Müller & Reisz (2006) (eureptiles and outgroups), following the "best practices" recommended by Parham et al. (2012).

Supplemental Table S2. Date ranges for Operational Taxonomic Units (OTUs) in Müller & Reisz (2006) (eureptiles and outgroups) used in a preliminary "practice". Dates derived from FossilWorks/the Paleobiology Database (PaleobioDB).

Supplemental Table S3. Summary statistics on morphology data matrix from Müller & Reisz (2006). Calculated with *BEASTmasteR* functions.

Supplemental Table S4. Comparison of summary statistics from the five Beast2 runs using tip dates derived from the Paleobiology Database.

Supplemental Table S5. Number of patterns that are unobservable in the $Mk_{parsinf}$ model, which excludes parsimony-uninformative characters (invariant and autapomorphic characters). The ascertainment-bias correction requires that the likelihood be calculated for each of these

patterns, which obviously becomes problematic when there are millions of such patterns. The calculations here assume unordered characters. This table duplicates Table 2, but is included in Supplemental Material for comparison. For ordered characters, see Supplemental Table S8.

Supplemental Table S6. Total number of possible patterns, determined by (# of states)$^{(\# \text{ of taxa})}$. "Inf" is not literally infinity, it just means the result exceeds R's numerical limit.

Supplemental Table S7. Fraction of the total number of possible patterns that are unobservable under M$k_{parsinf}$ ascertainment bias correction. The total number of possible patterns is determined by (# of states)$^{(\# \text{ of taxa})}$. For ordered characters, see Supplemental Table S10.

Supplemental Table S8. Number of patterns that are unobservable in the M$k_{parsinf}$ model, assuming ordered characters. For unordered characters, see Supplemental Table S5.

Supplemental Table S9. Total number of possible patterns, determined by (# of states)^(# of taxa). "Inf" is not literally infinity, it just means the result exceeds R's numerical limit.

Supplemental Table S10. Fraction of the total number of possible patterns that are unobservable under M$k_{parsinf}$ ascertainment bias correction, assuming ordered characters. For unordered characters, see Supplemental Table S7.

Supplemental Table S11. Comparison of summary statistics from the six Beast2 runs on a simulated "strict clock" dataset.

Supplemental Table S12. Comparison of summary statistics from the six Beast2 runs on a simulated "no clock" dataset.

MP tree
(branch lengths)

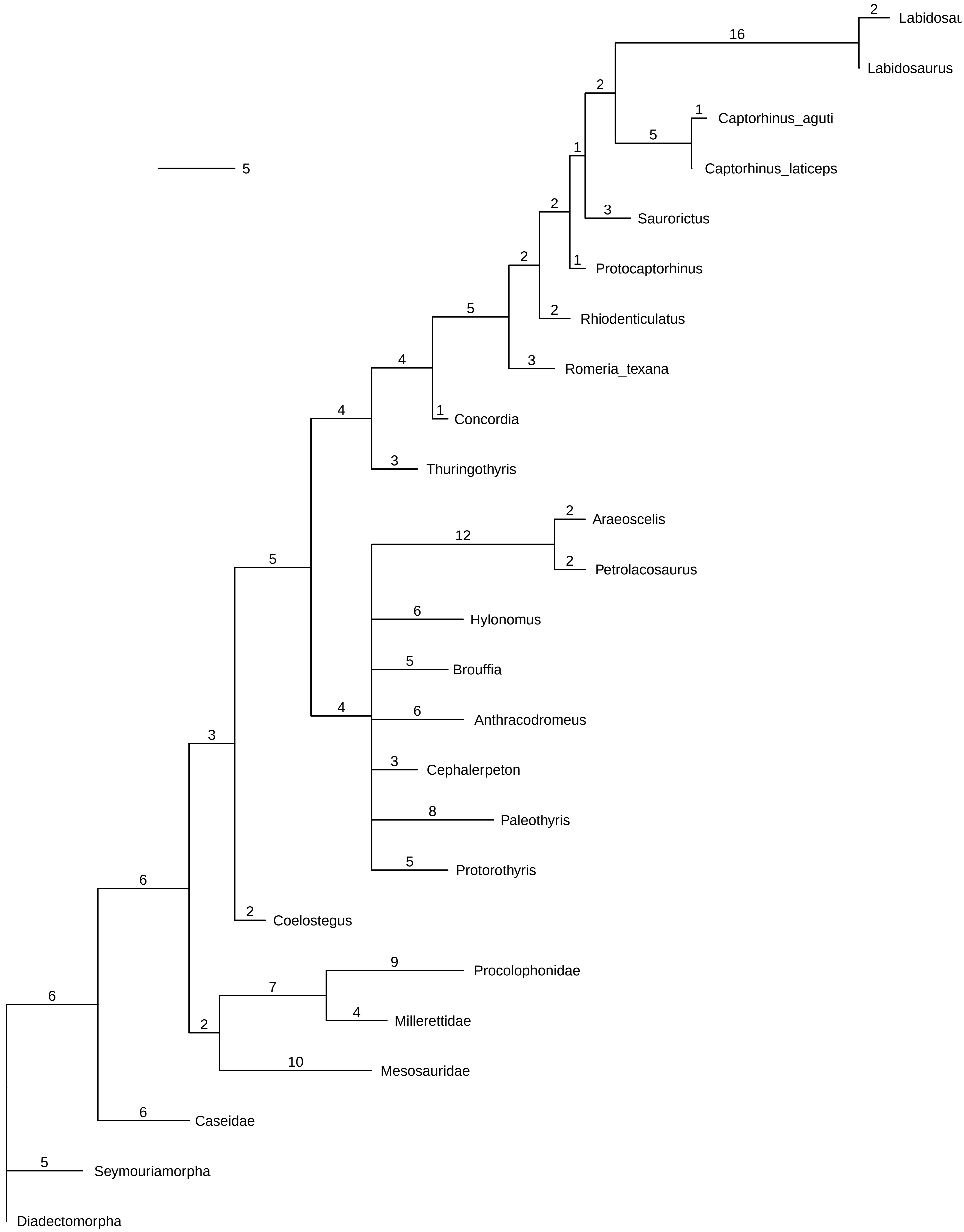

Supplemental Figure S1

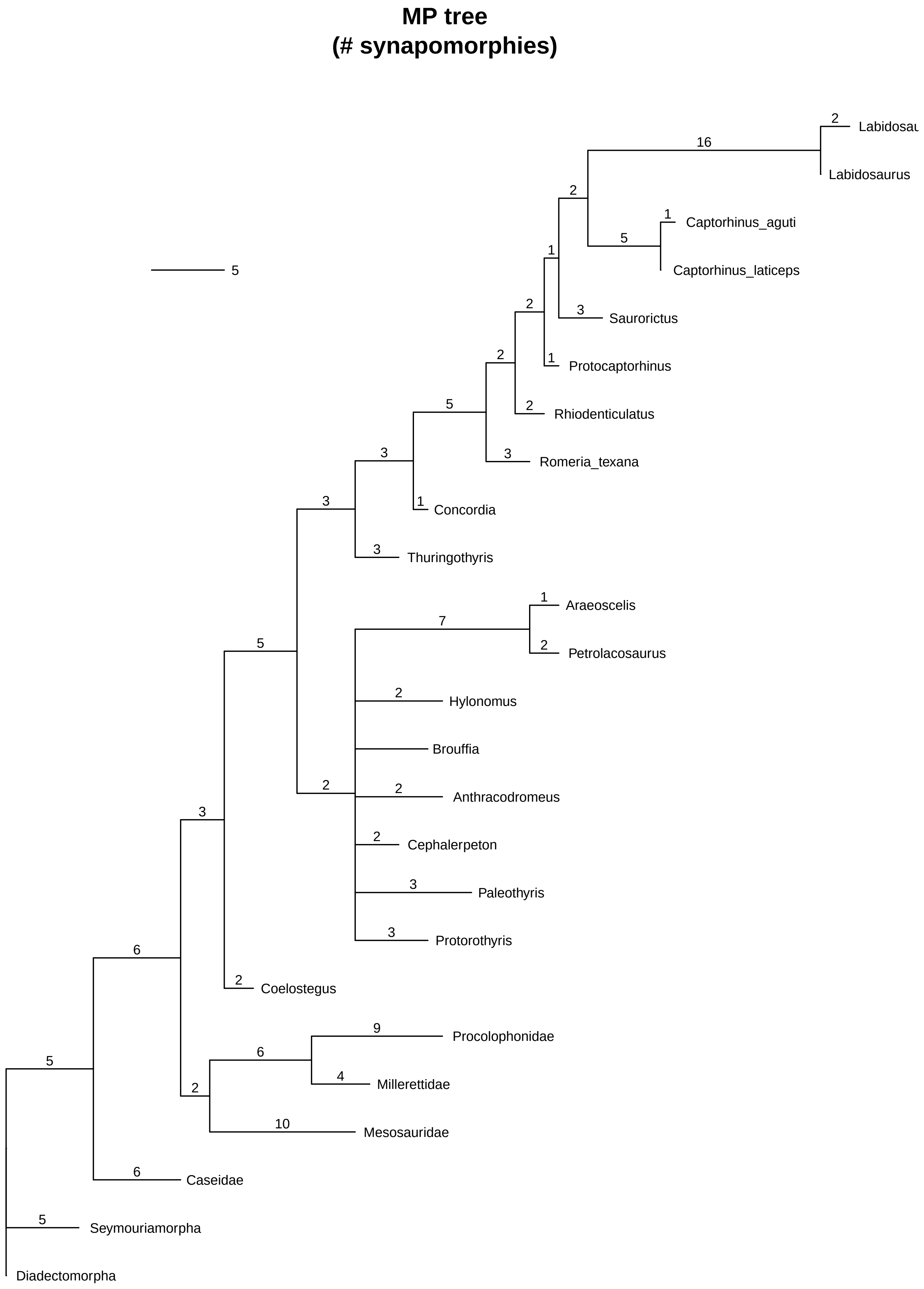

Supplemental Figure S2

MP tree
(absolute Bremer support/decay index)

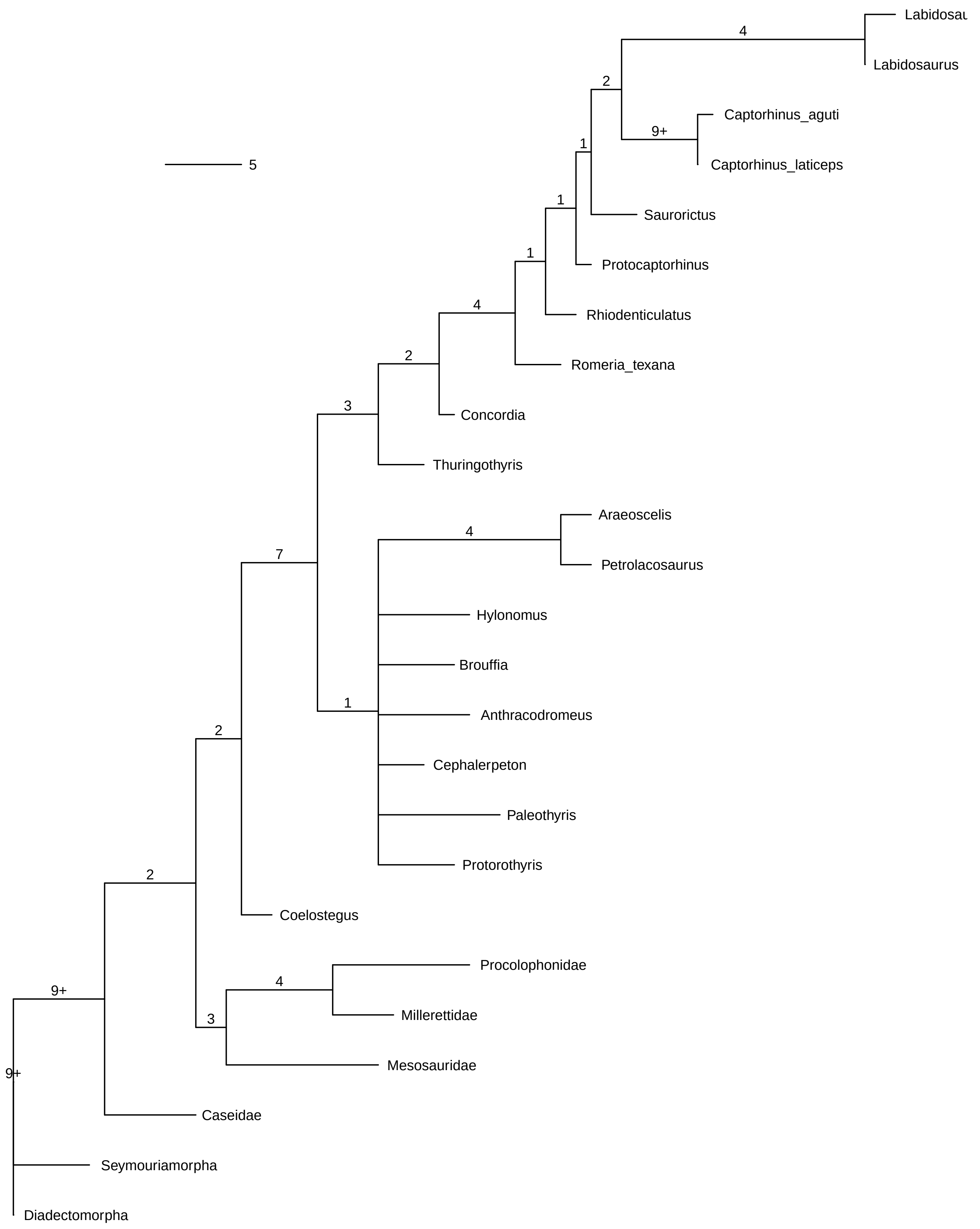

Supplemental Figure S3

MP tree
(relative Bremer support/decay index)

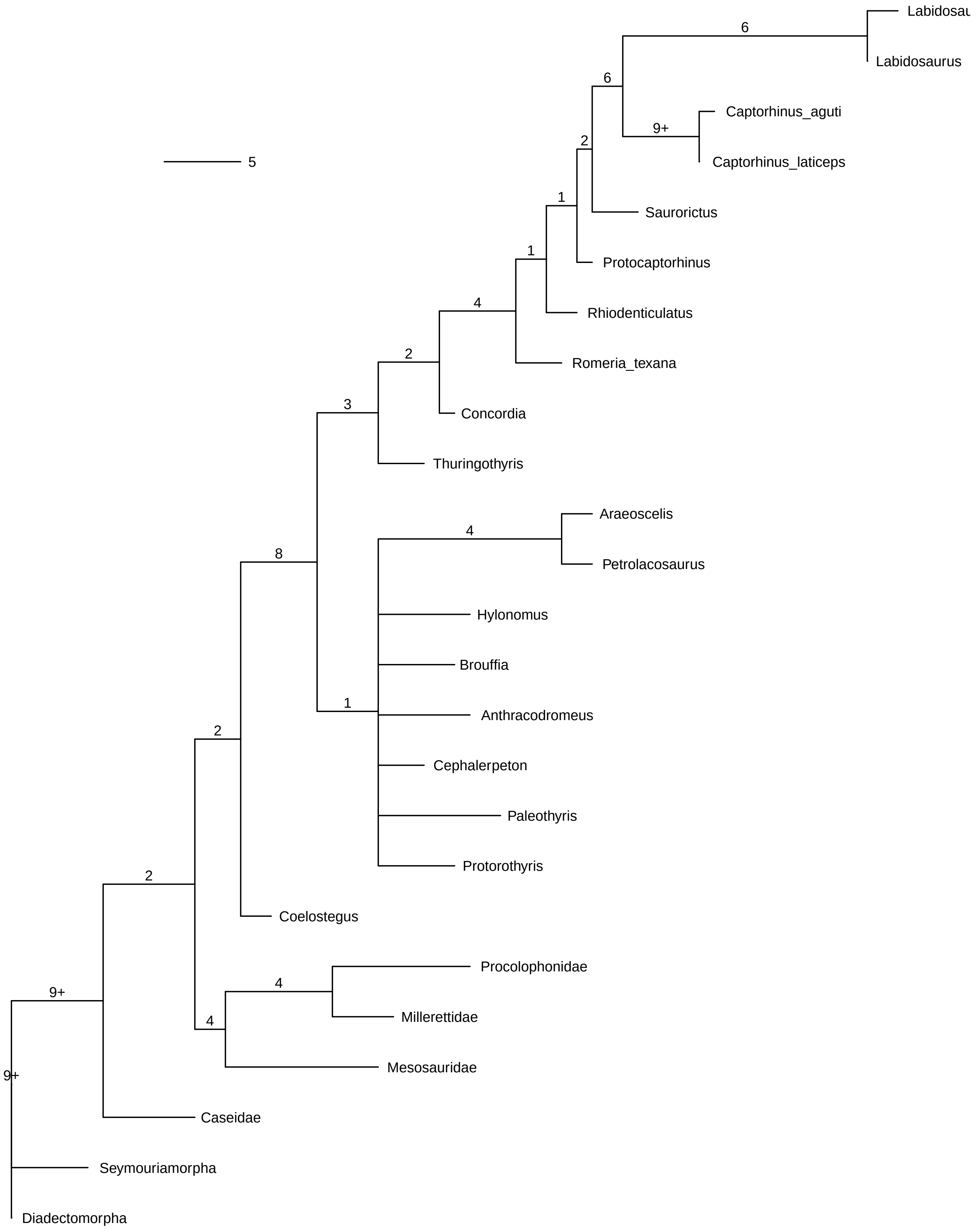

Supplemental Figure S4

MP tree
(bootstrap support/frequency)

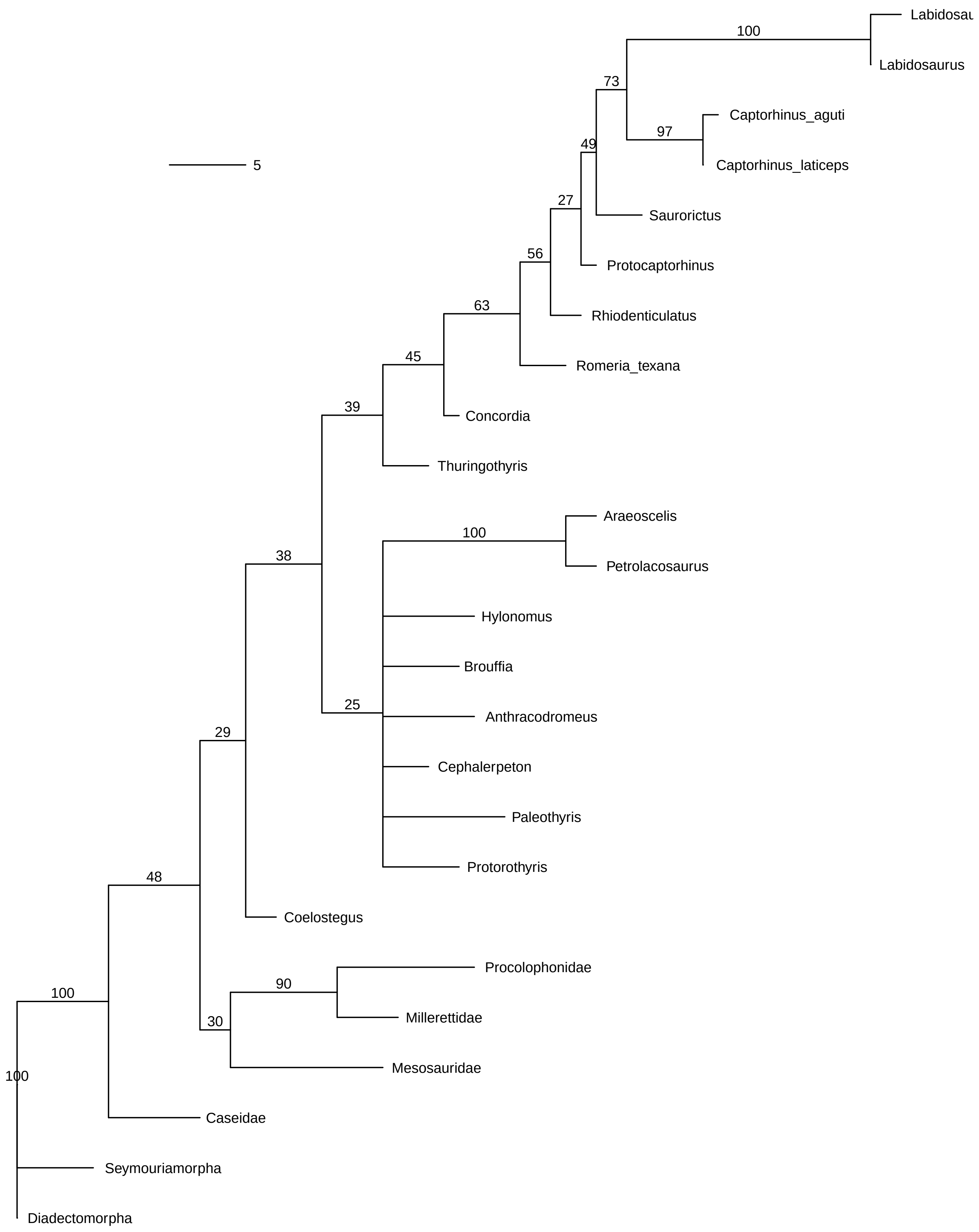

Supplemental Figure S5

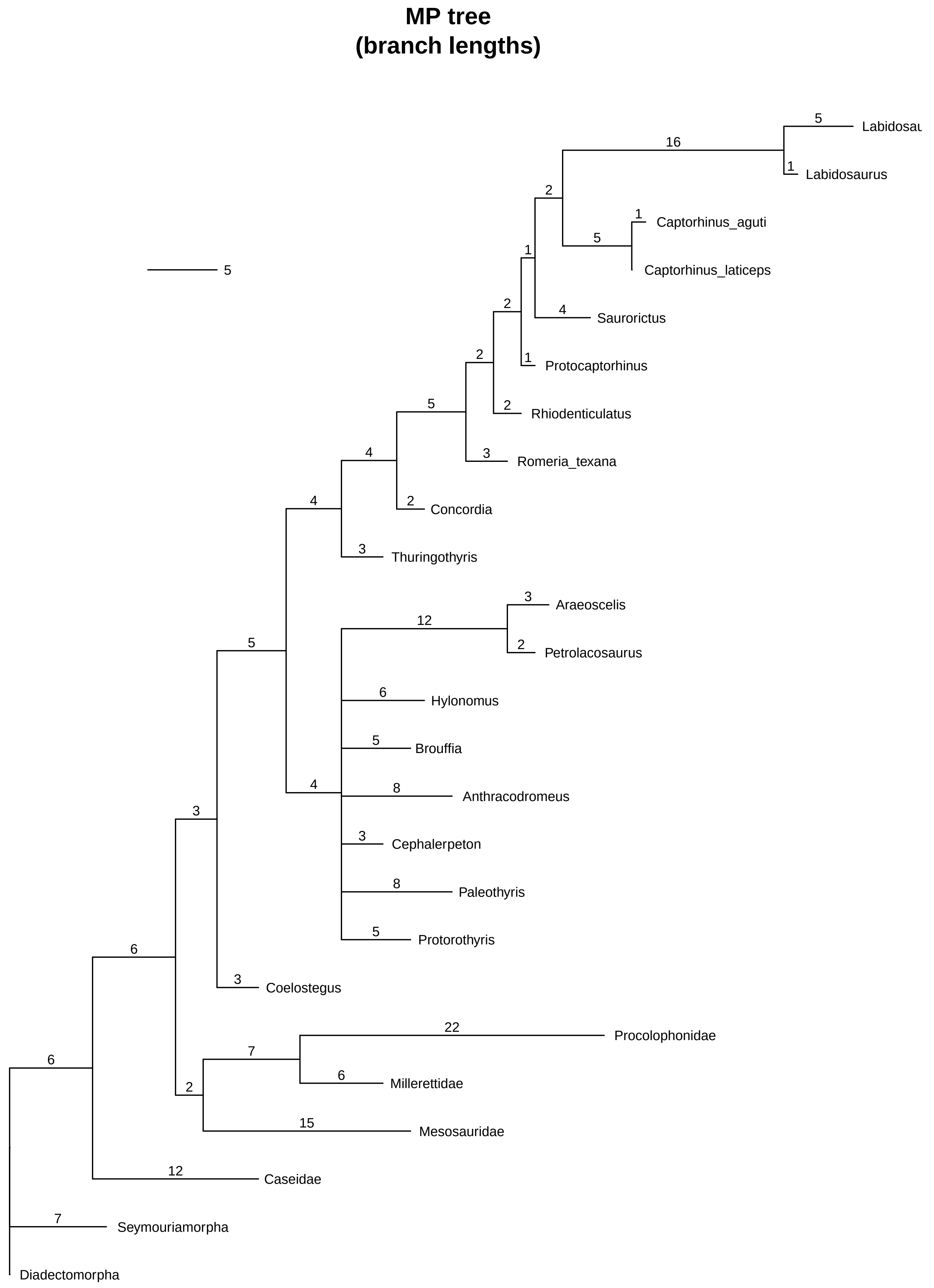

Supplemental Figure S6

MP tree
(# synapomorphies)

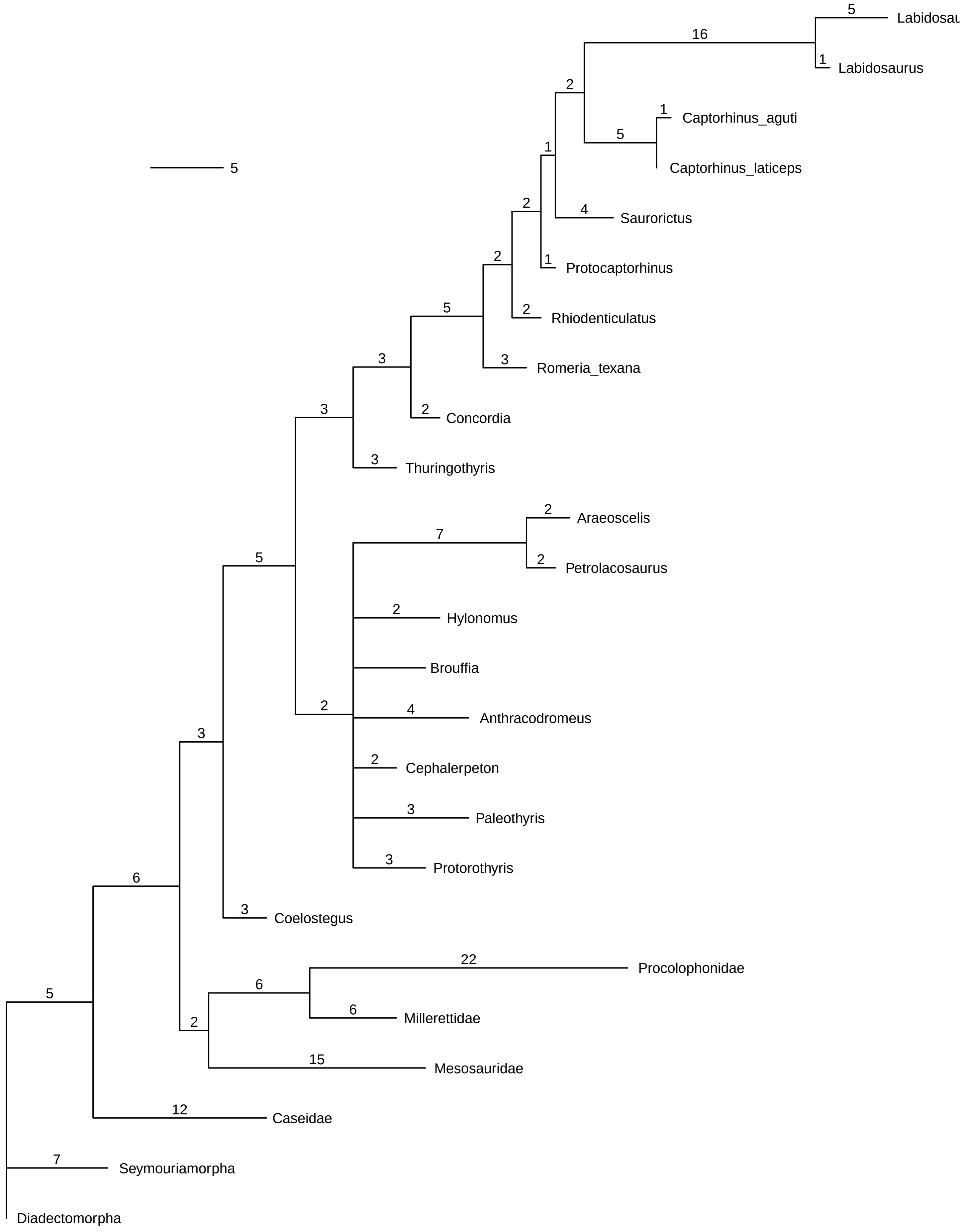

Supplemental Figure S7

MP tree
(absolute Bremer support/decay index)

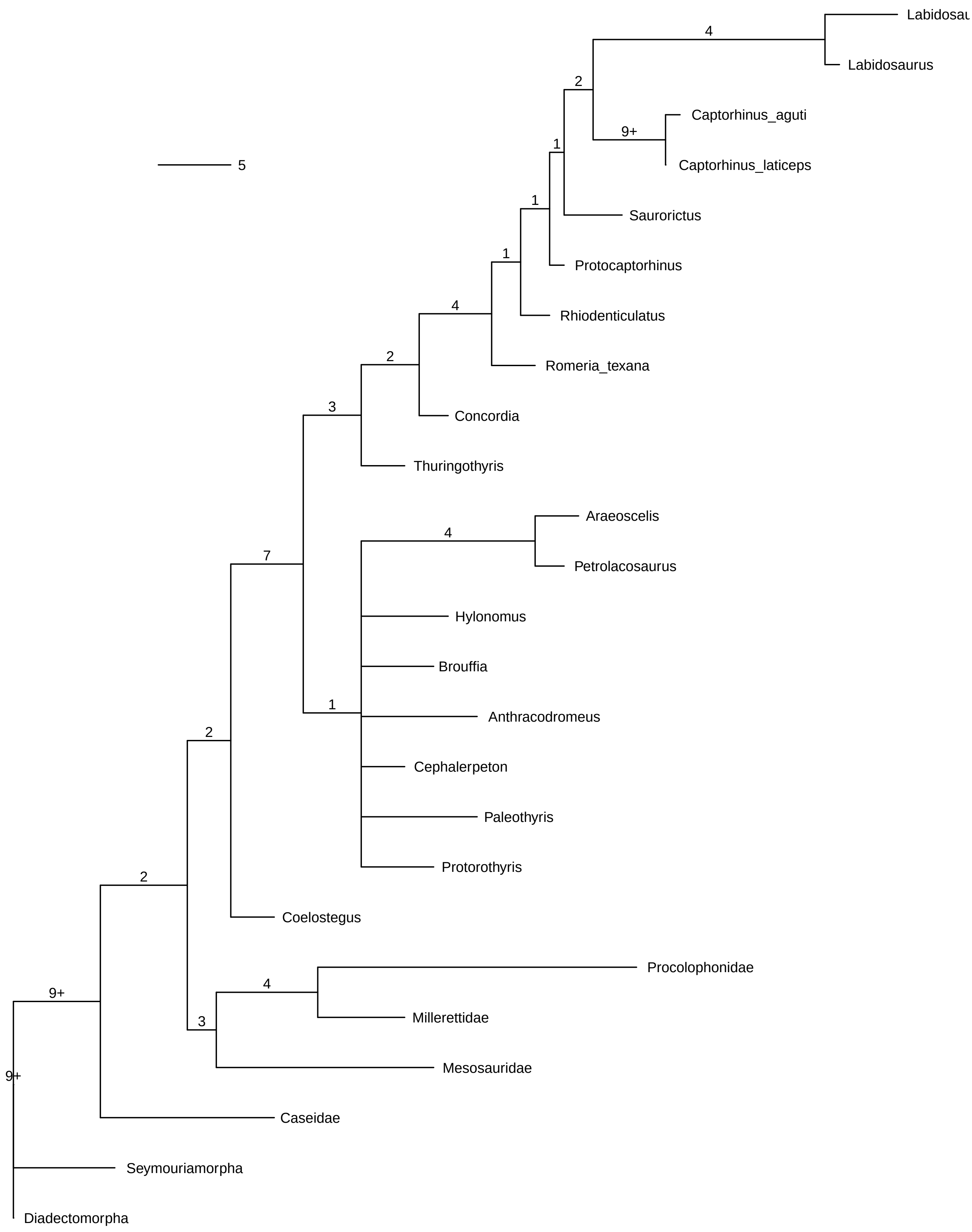

Supplemental Figure S8

MP tree
(relative Bremer support/decay index)

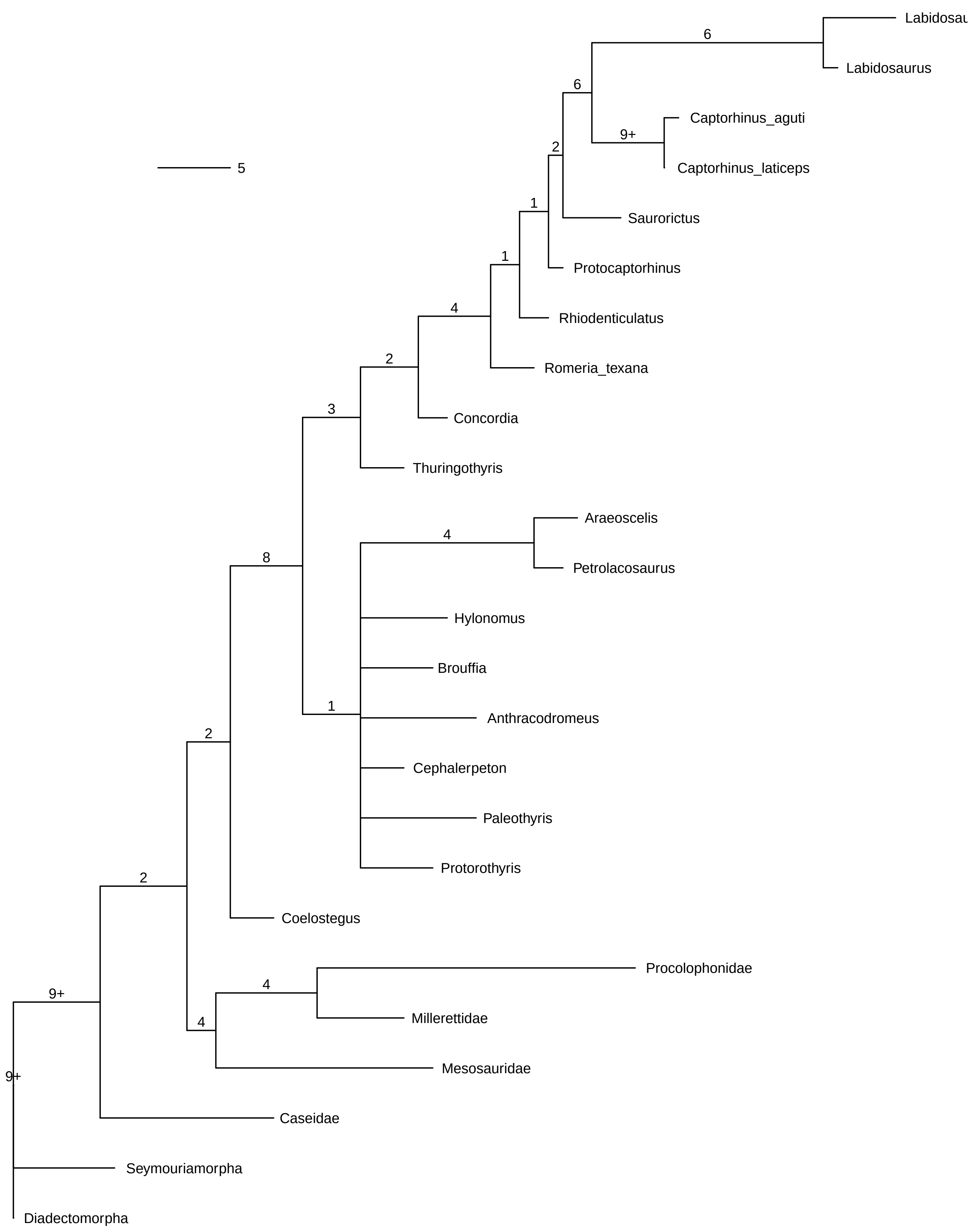

Supplemental Figure S9

MP tree
(bootstrap support/frequency)

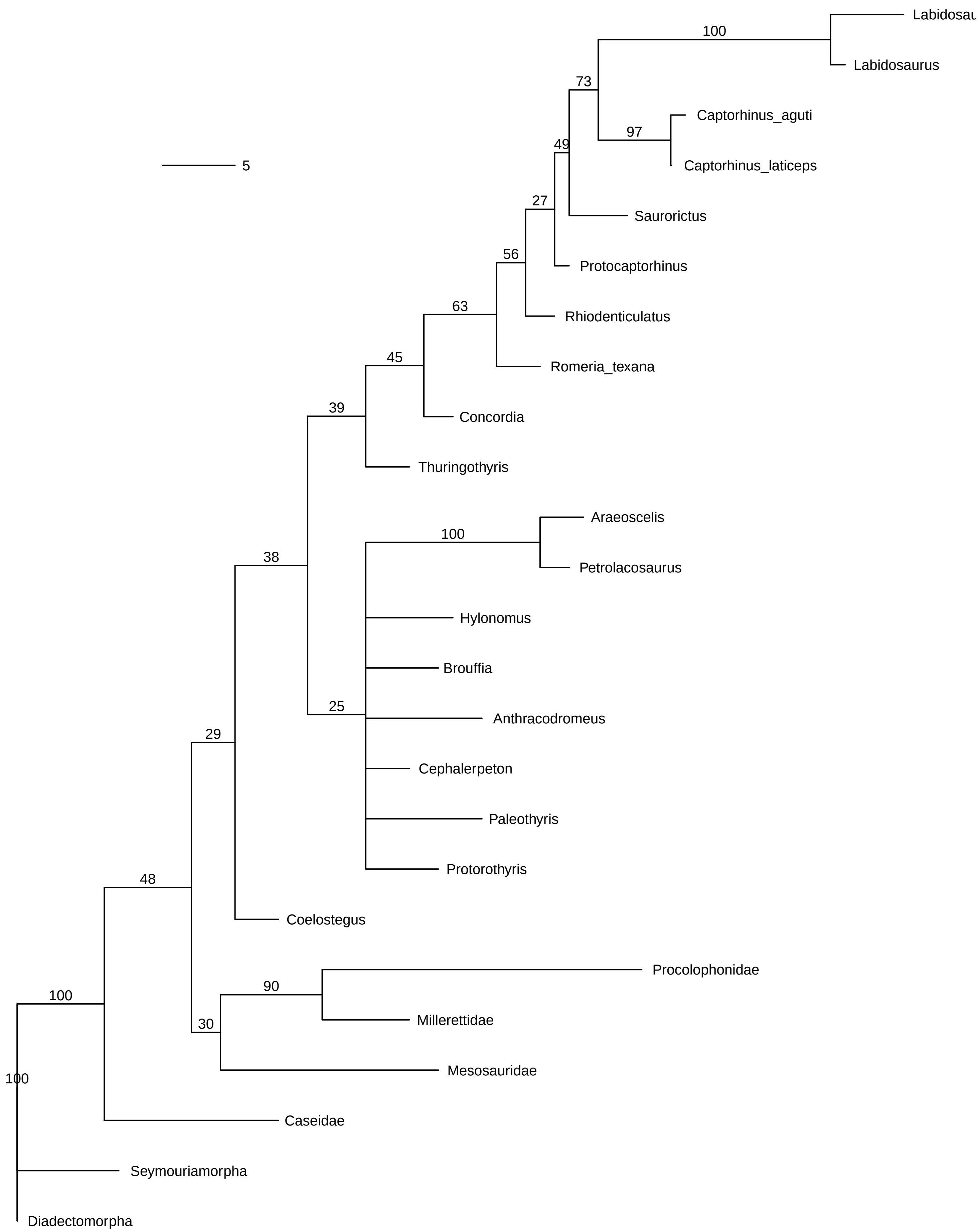

Supplemental Figure S10

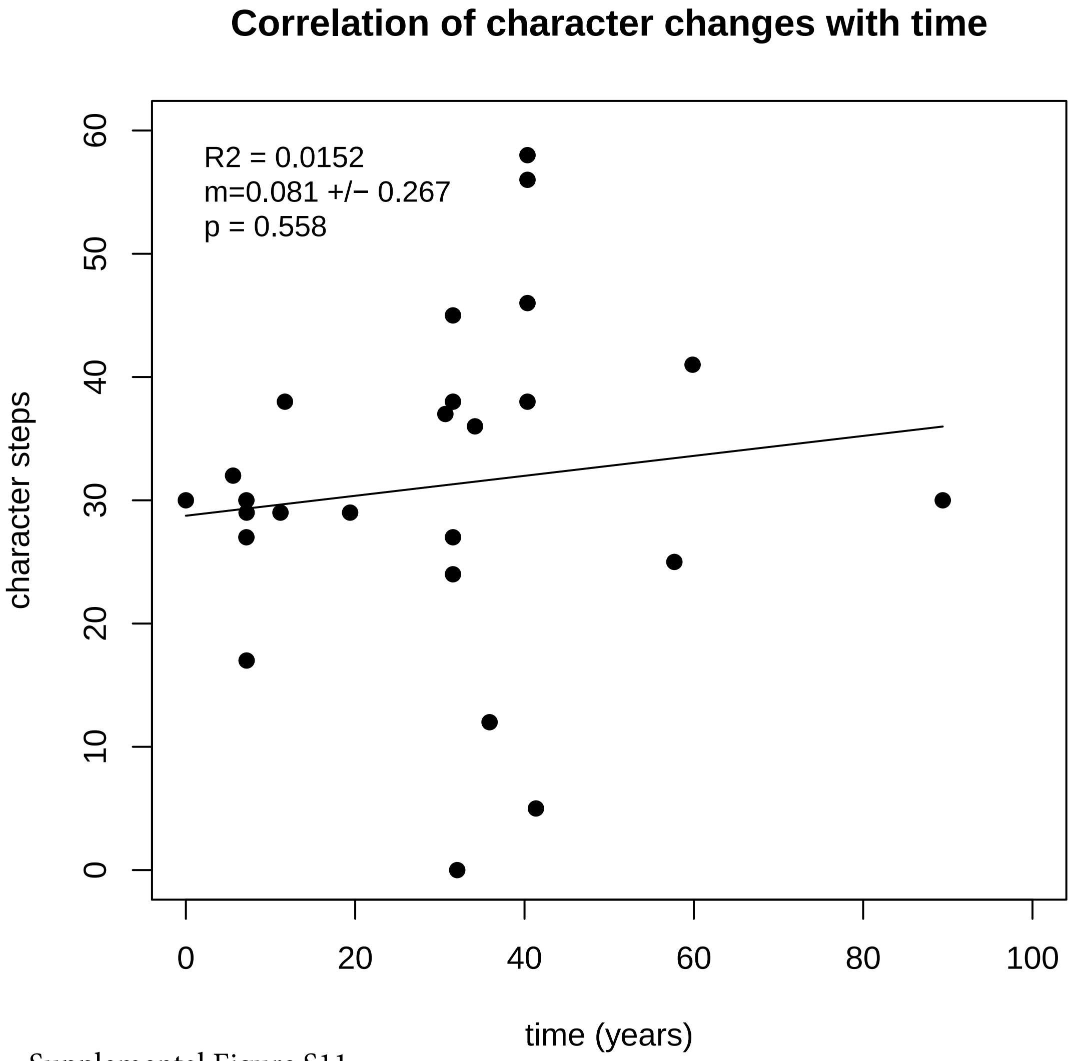

Supplemental Figure S11

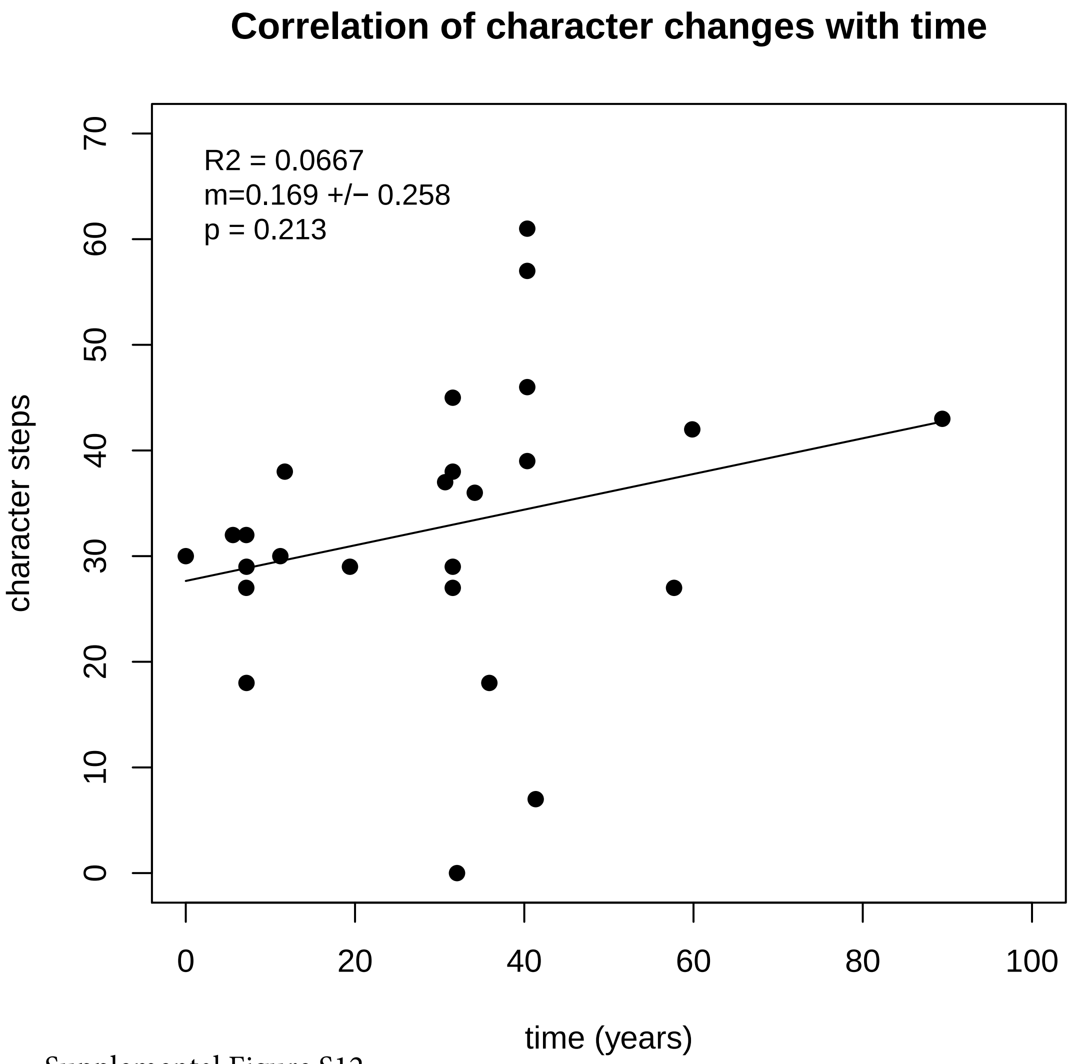

Supplemental Figure S12

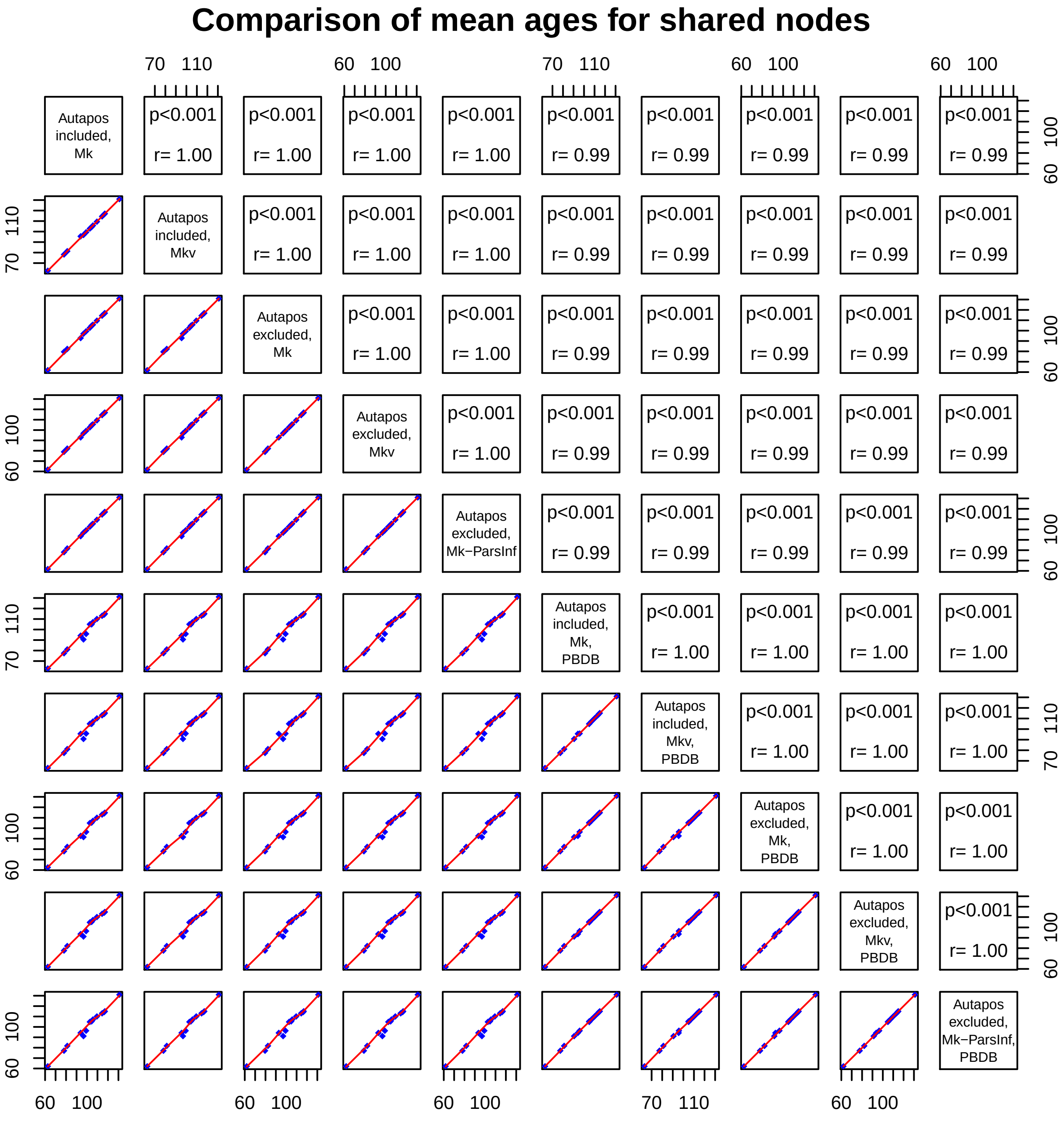

Supplemental Figure S13

Comparison of node age HPD widths

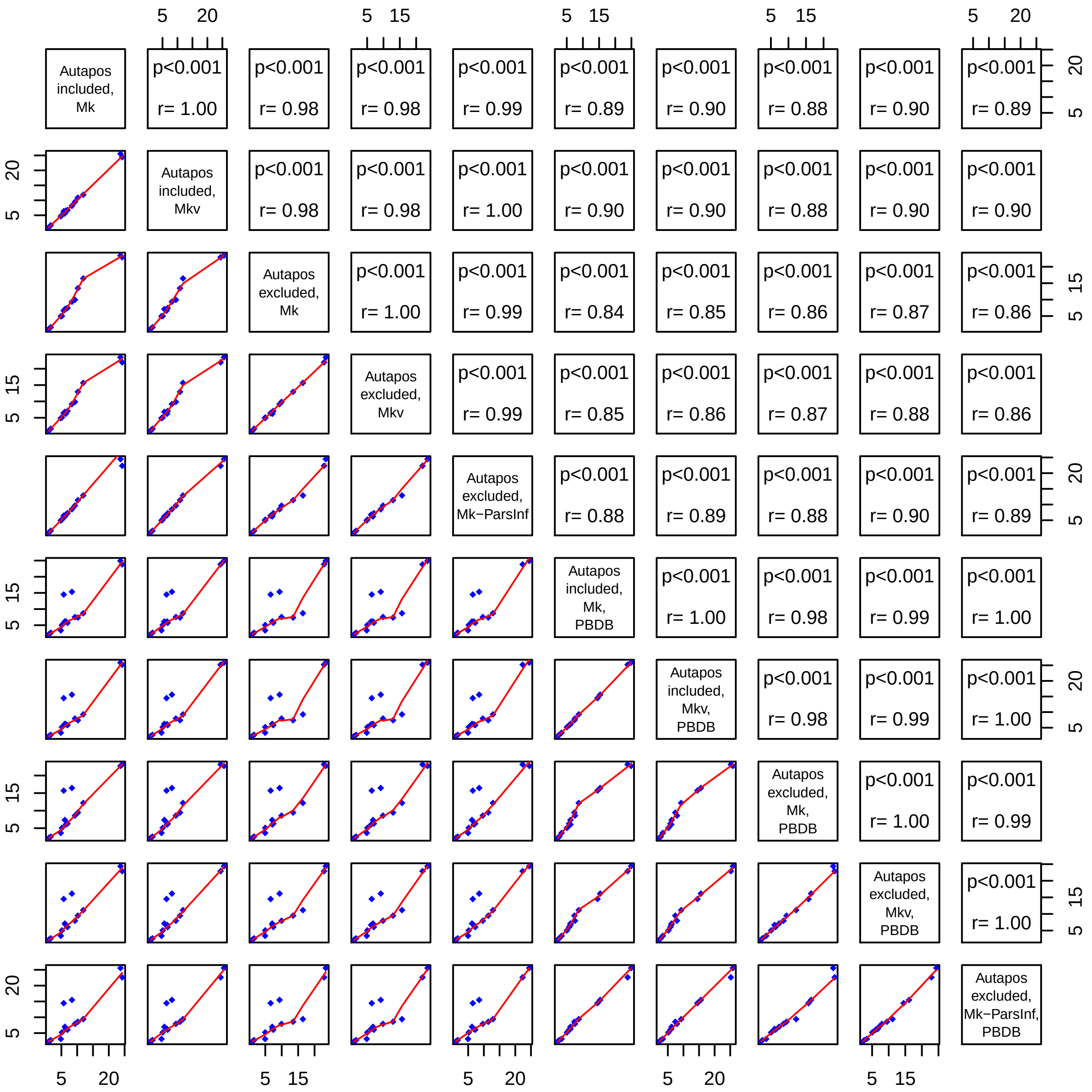

Supplemental Figure S14

Supp. Fig. S15

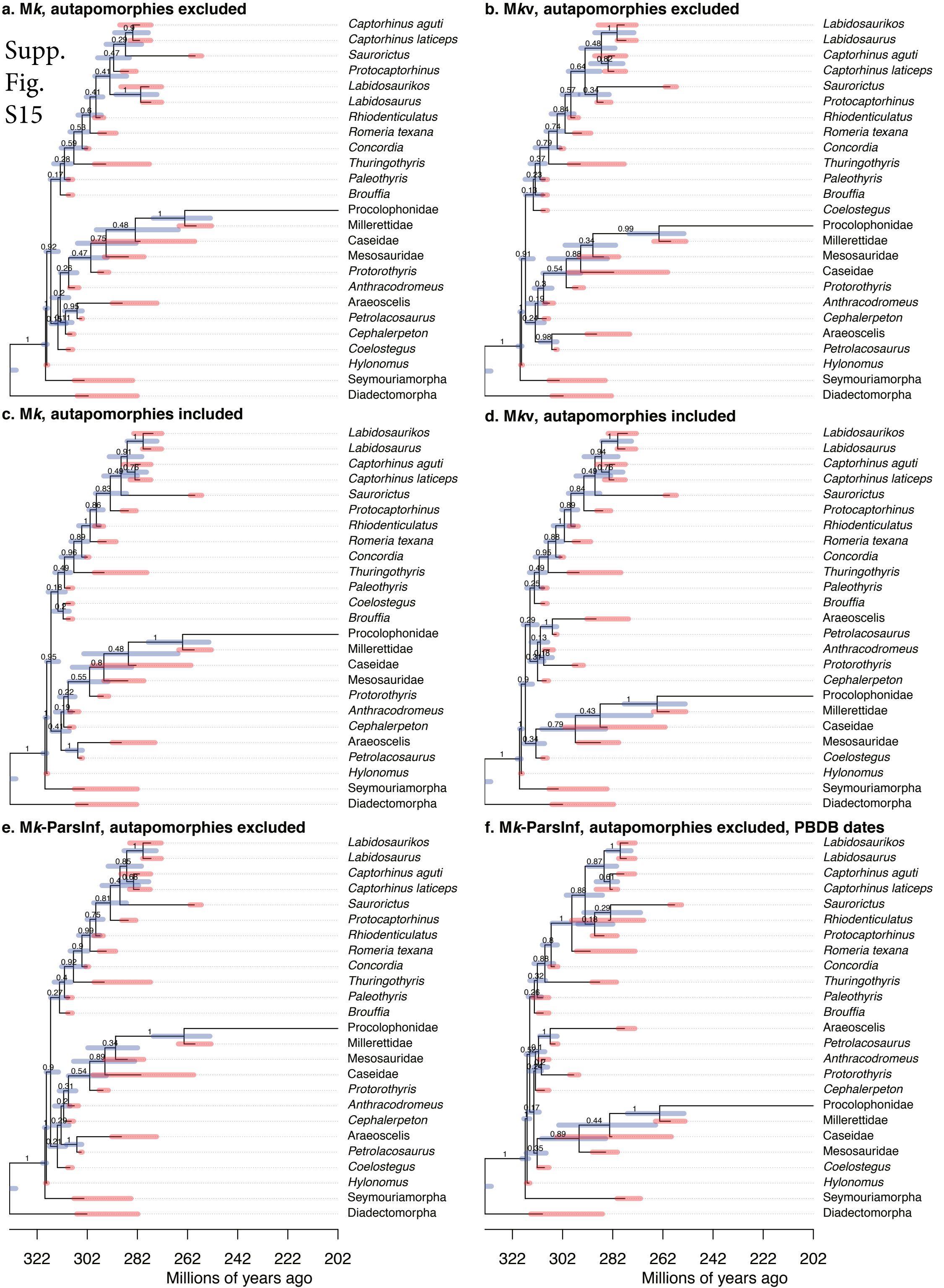
a. Mk, autapomorphies excluded
Captorhinus aguti
Captorhinus laticeps
Saurorictus
Protocaptorhinus
Labidosaurikos
Labidosaurus
Rhiodenticulatus
Romeria texana
Concordia
Thuringothyris
Paleothyris
Brouffia
Procolophonidae
Millerettidae
Caseidae
Mesosauridae
Protorothyris
Anthracodromeus
Araeoscelis
Petrolacosaurus
Cephalerpeton
Coelostegus
Hylonomus
Seymouriamorpha
Diadectomorpha
b. Mkv, autapomorphies excluded
Labidosaurikos
Labidosaurus
Captorhinus aguti
Captorhinus laticeps
Saurorictus
Protocaptorhinus
Rhiodenticulatus
Romeria texana
Concordia
Thuringothyris
Paleothyris
Brouffia
Coelostegus
Procolophonidae
Millerettidae
Mesosauridae
Caseidae
Protorothyris
Anthracodromeus
Cephalerpeton
Araeoscelis
Petrolacosaurus
Hylonomus
Seymouriamorpha
Diadectomorpha
c. Mk, autapomorphies included
Labidosaurikos
Labidosaurus
Captorhinus aguti
Captorhinus laticeps
Saurorictus
Protocaptorhinus
Rhiodenticulatus
Romeria texana
Concordia
Thuringothyris
Paleothyris
Coelostegus
Brouffia
Procolophonidae
Millerettidae
Caseidae
Mesosauridae
Protorothyris
Anthracodromeus
Cephalerpeton
Araeoscelis
Petrolacosaurus
Hylonomus
Seymouriamorpha
Diadectomorpha
d. Mkv, autapomorphies included
Labidosaurikos
Labidosaurus
Captorhinus aguti
Captorhinus laticeps
Saurorictus
Protocaptorhinus
Rhiodenticulatus
Romeria texana
Concordia
Thuringothyris
Paleothyris
Brouffia
Araeoscelis
Petrolacosaurus
Anthracodromeus
Protorothyris
Cephalerpeton
Procolophonidae
Millerettidae
Caseidae
Mesosauridae
Coelostegus
Hylonomus
Seymouriamorpha
Diadectomorpha
e. Mk-ParsInf, autapomorphies excluded
Labidosaurikos
Labidosaurus
Captorhinus aguti
Captorhinus laticeps
Saurorictus
Protocaptorhinus
Rhiodenticulatus
Romeria texana
Concordia
Thuringothyris
Paleothyris
Brouffia
Procolophonidae
Millerettidae
Mesosauridae
Caseidae
Protorothyris
Anthracodromeus
Cephalerpeton
Araeoscelis
Petrolacosaurus
Coelostegus
Hylonomus
Seymouriamorpha
Diadectomorpha
f. Mk-ParsInf, autapomorphies excluded, PBDB dates
Labidosaurikos
Labidosaurus
Captorhinus aguti
Captorhinus laticeps
Saurorictus
Rhiodenticulatus
Protocaptorhinus
Romeria texana
Concordia
Thuringothyris
Paleothyris
Brouffia
Araeoscelis
Petrolacosaurus
Anthracodromeus
Protorothyris
Cephalerpeton
Procolophonidae
Millerettidae
Caseidae
Mesosauridae
Coelostegus
Hylonomus
Seymouriamorpha
Diadectomorpha
322
302
282
262
242
222
202
Millions of years ago
322
302
282
262
242
222
202
Millions of years ago

Supplemental Table S1. Date ranges for Operational Taxonomic Units (OTUs) in Müller & Reisz (2006) (eureptiles and outgroups), following the "best practices" recommended by Parham et al. (2012).

Operational Taxonomic Units (OTUs)	Dates (Ma) max	Dates (Ma) min	Justification
Diadectomorpha	306	272.3	The diadectid *Desmatodon* is the oldest unambiguous diadectomorph, with occurrences dated to the Missourian (Kissel & Reisz 2004); the lower boundary of this unit is 306 Ma according to the 2012 Geologic Timescale (2012 GTS - Gradstein et al. 2012). Several diadectid taxa are present in the Leonardian/Kungurian (Kissel & Reisz 2004; Kissel 2010); the upper boundary of these units is 272.3 Ma in the 2012 GTS.
Seymouriamorpha	307.1	251.9	The potential oldest seymouriamorph is *Utgenia* from the Upper Carboniferous-Lower Permian of Russia (Klembara & Ruta 2004). The age of this taxon is not well-constrained, so we conservatively give it a maximum age of Upper Pennsylvanian, whose lower boundary is dated to 307 ± 0.1 Ma in the 2012 GTS. The youngest seymouriamorph is *Karpinskiosaurus* (=*Kotlassia*) from Russia (Klembara 2011), whose stratigraphic range extends to near or the end of the Permian Period (Benton et al. 2004), which is dated to 251.9 ± 0.02 Ma by Burgess et al. (2014).
Caseidae	302	257	The recently described *Eocasea* is the oldest-known caseid and Virgilian/Stephanian in age (Reisz & Fröbisch 2014). The upper boundary of the Stephanian is between 302-300 Ma in the 2012 GTS. The youngest well-dated caseid is *Ennatosaurus* from Russia, which is Tatarian (Maddin et al. 2008). Reisz et al. (2011) *Ruthenosaurus* and *Euromycter* could be as young as early Lopingian, but new radioisotopic ages from the nearby correlative Lodève Basin make an Artinksian-Kungurian age more likely (Michel et al. 2015). The Tartarian is 269-257 Ma according to the 2012 GTS.
Mesosauridae	295.9	278.7	All known mesosaurid occurrences are Sakmarian-Artinskian in age (Modesto 1999, 2006, 2010; Piñeiro et al. 2012), which gives an age range of 295.5 ± 0.4 Ma to 279.3 ± 0.6 Ma according to the 2012 GTS.
Millerettidae	265.5	251.9	The oldest millerettid is *Broomia* from the *Tapinocephalus* Assemblage Zone of southern Africa (Cisneros et al. 2008); the minimum age of this biostratigraphic zone is constrained by U-Pb dates (Rubidge et al. 2013; Day et al. 2015), but its maximum age is not. Therefore, we are conservative in using the lower boundary of the Capitanian as a maximum age for this taxon; this is dated to 265.1 ± 0.4 Ma in the 2012 GTS. The youngest millerettids occur in the Dicynodon Assemblage Zone (Rubidge 2005; Cisneros et al. 2008), the upper boundary of which is thought to coincide with the Permian-Triassic boundary. This is dated to 251.9 ± 0.02 Ma (Burgess et al. 2014).
Procolophonidae	260.3	201.6	The maximum age for Procolophonidae is difficult to constrain, because fossils are either phylogenetically well-constrained but poorly dated (*Pintosaurus*), or poorly-constrained phylogenetically but relatively-well dated (*Spondylolestes* and *Kinelia*) (Cisneros 2008). Nonetheless, these data collectively suggest a Late Permian origin for procolophonids, which is consistent with the Late Permian appearance of their sister clade Owenettidae. Thus, we conservatively use the base of the Wuchiapingian as the maximum age of Procolophonidae, which is dated to 259.9 ± 0.4 Ma in the 2012 GTS. Procolophonids went extinct at or near the end-Triassic mass extinction, which has been dated to 201.6 Ma (Blackburn et al. 2013).
Romeria_texana	297	290	*Romeria* is from the Archer City Formation of Texas (Hook 1989; Hentz 1989), which is equivalent to the Moran through Santa Ana Branch Shale formations to the south (Hentz 1989). This series of units is upper Asselian to Sakmarian in age (Wardlaw 2005), which spans 297 Ma to 290.1 ± 0.1 Ma based on the 2012 GTS.
Protocaptorhinus	288	282	This taxon is known from the Petrolia Formation of Texas; Hook (1989) listed occurrences from the Nocona and Waggoner Ranch formations, but these have not been described and we do not consider their stratigraphic range here. The Petrolia is equivalent to the Elm Creek, Jagger Bend, and Valera Shale formations further south (Hentz 1989), which are middle Artinskian in age (Wardlaw 2005). Based on the 2012 GTS, this gives an age range of approximately 288 to 282 Ma.

Rhiodenticulatus	299.1	295.1		All known specimens of this taxon are from a single locality (Camp Quarry) in the Arroyo del Agua Formation (Cutler Group) of New Mexico (Lucas et al. 2005a,b). Although the placement of the Carboniferous-Permian boundary in this section is controversal, all recent authors agree that the Camp Quarry is lowermost Permian in age. We conservatively consider it somewhere within the Asselian, which spans 298.9 ± 0.2 Ma to 295.5 ± 0.4 Ma in the 2012 GTS.
Captorhinus_laticeps	285	276		The type of *C. laticeps* is from the Waggoner Ranch (=Clyde) Formation of west Texas (Heaton 1979), which correlates to the Bead Mountain through Lueders formations further south (Hentz 1989). These units are dated to the upper Artinskian through lower Kungurian (Wardlaw 2005), which is between 285 and 276 Ma in the 2012 GTS. Referred material from the Wellington Formation of Oklahoma (Heaton 1979; Modesto 1998) is thought to be lower Leonardian in age (Sawin et al. 2008), which is within the same age range. Material from the Richards Spur locality that Heaton (1979) assigned to *C. laticeps* has subsequently been referred to *C. aguti* (Bolt 1980; Modesto 1998).
Captorhinus_aguti	291	276		*C. aguti* is best known by abundant remains recovered from the Richards Spur locality in Oklahoma, for which a U-Pb speleothem age of 289 ± 0.68 Ma was recently published (Woodhead et al. 2010). Potential open-system behavior (e.g., lead loss) is difficult to fully ascertain with such carbonate samples, and the duration of deposition at Richards Spur is unclear, so we prefer to take a conservative approach and consider an age range for this taxon that is larger than the analytical uncertainty for this date. Most of the material reported from Texas (Fox & Bowman 1966) has either been re-assigned to other taxa (Heaton 1979; Modesto 1998) or has not been re-evaluated since. Modesto (1998) assigned specimens from the Lueders Formation in Texas to *C. aguti*; Wardlaw (2005) assigned this unit to the lower Kungurian, providing a minimum age for this taxon of 276 Ma using the 2012 GTS.
Labidosaurus	279.9	271.8		Known specimens of *Labidosaurus* are from the lowermost portion of the undivided Clear Fork Group of west Texas (Williston 1917; Sumida 1987, 1989; Modesto et al. 2007). This unit is Kungurian in age (Wardlaw 2005), but lacks more precise age constraints. The Kungurian spans 279.3 ± 0.6 to 272.3 ± 0.5 Ma in the 2012 GTS.
Labidosaurikos	291	271.8		The only known specimen of *Labidosaurikos* is from the Hennessey Formation of Oklahoma (Dodick & Modesto 1995). Few non-vertebrate biostratigraphic age constraints are available for this formation. Based on the vertebrate assemblage, Olson (1967, 1970) proposed that the Hennessey was correlative with the Clear Fork Group (=Vale & Choza formations) in Texas. See the age justification of *Labidosaurus* above for the age range of the Clear Fork Group. In contrast, the Hennessey also shares several vertebrate taxa with the Richards Spur locality in Oklahoma, which was recently dated to 289 ± 0.68 Ma (see age justification for *Captorhinus aguti*). Therefore, we take the conservative approach and assign an age range to *Labidosaurikos* that encapsulates the age range for both potential correlations.
Saurorictus	260.73	255.93		*Saurorictus* is from the *Tropidostoma* Assemblage Zone of the Karoo Basin, South Africa (Modesto & Smith 2001). Recent high-resolution U-Pb zircon ages bracket this zone to between 260.41 ± 0.32 and 256.25 ± 0.32 Ma (Rubidge et al. 2013).
Concordia	302.25	301		All known material of *Concordia* is from the Hamilton Quarry in the Calhoun Shale, Kansas (Müller & Reisz 2005). This unit is assigned to the *Streptognathodus virgilicus* conodont zone (Ritter 1995), which is dated to between 302.25 and 301 Ma in the 2012 GTS.
Protorothyris	297	293		The type species of *Protorothyris*, *P. archeri*, is from the lower Archer City (=Moran) Formation of west Texas (Price 1937; Clark & Carroll 1973). This unit correlates to the Moran and Sedwick formations further south (Hentz 1989), which are upper Asselian to lower Sakmarian in age (Wardlaw 2005). Based on the 2012 GTS, this suggests an age range of 297-293 Ma for *P. archeri*. The referred species *P. morani* is known only from the Washington Formation of the Dunkard Group in West Virginia (Clark & Carroll 1973). This unit is poorly dated; the best age constraints come from insect and vertebrate biogeography, which suggest a broadly Asselian-Sakmarian age for the formation (Schneider et al. 2013; Lucas 2013). The closest conodont age constraint is from the Ames Limestone of the underlying Conemaugh Group, which is assigned to the *Idiognathus simulator* zone (Heckel et al. 2011), suggesting only that the Dunkard Group is younger than lower Gzhelian (i.e., <302 Ma based on the 2012 GTS).

Paleothyris	309.5	307.5		All known specimens were found above the Lloyd Cove/Lower Bonar Coal in the Morien Group of Nova Scotia (Carroll 1969), which is dated to the Westphalian D regional sub-stage (Zodrow & Cleal 1985; Gibling & Bird 1994). This sub-stage has an age range of 309.5 to 307.5 Ma in the 2012 GTS.
Cephalerpeton	309	307		The only specimen of *Cephalerpeton* is from the Francis Creek Shale of Mazon Creek, Illinois (Carroll & Baird 1972). Based on conodont biostratigraphy, this unit correlates to the Verdigris cyclothem in the mid-continent (Heckel 2013), which is assignable to the *Neognathodus roundyi* conodont zone (Barrick et al. 2013). The age of this zone is 309 to 307 Ma in the 2012 GTS.
Anthracodromeus	309	305		This taxon is from the Upper Freeport Coal of the Allegheny Group in Ohio (Carroll & Baird 1972). The presence of the conodonts *Idiognathodus* and *Neognathodus roundyi* in the underlying Washingtonville Shale (Sturgeon & Youngquist 1949; Merrill 1972) indicate a maximum age of upper Desmoinesian (Barrick et al. 2013) for the Upper Freeport Coal. The lowermost conodont-bearing strata of the overlying Conemaugh Group are assigned to the *Idiognathus cancellosus* zone (Heckel et al. 2011), which provides a minimum age of lower Missourian (Barrick et al. 2013) for the Freeport. Thus, we consider the age range for *Anthracodromeus* to be upper Desmoinesian to lower Missourian, with a numerical age of 309 to 305 Ma based on the 2012 GTS (see age justification for Cephalerpeton for age of the *N. roundyi* zone).
Brouffia	309.5	307.5		*Brouffia* is known from a single specimen from the Nýřany coal measures of the Czech Republic (Carroll & Baird 1972). This unit has been dated to the Westphalian D regional sub-stage based on palynomorph biostratigraphy (Kalibová-Kaiserová 1967; Kalibová 1989; Bek 1995), which is consistent with aquatic vertebrate biostratigraphic correlations (Zajíc 2000). See *Paleothyris* age justification above for Westphalian D numerical age.
Coelostegus	309.5	307.5		The single specimen of *Coelostegus* is also from Nýřany (Carroll & Baird 1972); see *Brouffia* age justification.
Hylonomus	318.5	317.5		All known specimens of *Hylonomus* are from the Joggins Formation of Nova Scotia (Carroll 1964), which has been dated using palynomorph biostratigraphy to the Langsettian regional stage (Dolby 1991, 2003; Calder et al. 2005). Using the 2012 GTS, this dates the formation to between 318.5 and 317.5 Ma.
Thuringothyris	299.1	271.8		*Thuringothyris* is represented by several specimens from the Bromacker Quarry of the Tambach Formation in Germany (Boy & Martens 1991; Müller et al. 2006). This site has traditionally been dated to the Wolfcampian because it shares the vertebrate taxa *Seymouria*, *Diadectes*, and *Dimetrodon* with Lower Permian strata in western North America (e.g., Sumida et al. 1996, 1998; Berman et al. 1998, 2001). However, it's worth noting that the only shared species between Germany and North America is *Seymouria sanjuanensis*, which is only known from the poorly-dated units of the upper Cutler Group - the Organ Rock Shale in Utah and Arroyo del Agua Formation in New Mexico (e.g., Vaughn 1966; Berman et al. 1987). Furthermore, all three genera have been reported from the Clear Fork Group in Texas (e.g., Romer 1928; Olson 1958), and both *Diadectes* and *Dimetrodon* are known from the Wellington Formation of Oklahoma (e.g., Olson 1967); both of these units could be as young as Kungurian in age (see above) and thus younger than Wolfcampian. Schneider & Werneburg (2006) assigned the Tambach Formation to the lower Artinskian because of the presence of the insect *Moravamylacris kukalovae*, but this biostratigraphic scheme is problematic even regionally in Europe (e.g., Michel et al. 2015). Given the uncertainties in the proposed correlations, we suggest a conservative approach, namely, that the Bromacker Quarry could be Wolfcampian or Leonardian in age (i.e., Asselian to Kungurian). As such we assign a large age range of 298.9 ± 0.2 to 272.3 ± 0.5 Ma to *Thuringothyris*.
Petrolacosaurus	304.3	303.6		All specimens of *Petrolacosaurus* are from the Rock Lake Shale of the Stanton Limestone in Kansas (Peabody 1952). This unit is assigned to the *Streptognathus firmus* conodont zone (Ritter 1995); in the 2012 GTS this zone is dated to between 304.3 and 303.7 ± 0.1 Ma.

Araeoscelis	292	271.8		A variety of specimens of this taxon have been collected from the Nocona (=Admiral) Formation, Petrolia (=Belle Plains) Formation, and lowermost Clear Fork Gorup (=Arroyo Fm) in Texas (Vaughn 1955; Reisz et al. 1984). These units correlate with the Coleman Junction through lower Cleark Fork Group further south (Hentz, 1989) which are assigned an upper Sakmarian through Kungurian age (Wardlaw 2005). Based on the 2012 GTS, this gives an age range of 292 to 272.3 ± 0.5 Ma.

References Cited in Supplemental Table 6

Barrick, J. E., Lambert, L. L., Heckel, P. H., Rosscoe, S. J., and Boardman, D. R., 2013, Midcontinent Pennsylvanian conodont zonation: Stratigraphy, v. 10, no. 1-2, p. 55-72.

Bek, J., 1995, Upper Carboniferous miospores near Slaný, Kladno Basin, part III - summary of the Ři-21, Ři-22, Ři-23 and Ři-24 boreholes: Věstnik Českého Geologického Ústavu, v. 70, no. 3, p. 53-66.

Benton, M. J., Tverdokhlebov, V. P., and Surkov, M. V., 2004, Ecosystem remodelling among vertebrates at the Permian-Triassic boundary in Russia: Nature, v. 432, p. 97-100.

Berman, D.S., Reisz, R. R., and Eberth, D. A., 1987, *Seymouria sanjuanensis* (Amphibia, Batrachosauria) from the Lower Permian Cutler Formation of north-central New Mexico and the occurrence of sexual dimorphism in that genus question: Canadian Journal of Earth Sciences, v. 24, p. 1769–1784.

Berman, D. S., Sumida, S. S., and Martens, T., 1998, *Diadectes* (Diadectomorpha: Diadectidae) from the early Permian of central Germany, with description of a new species: Annals of Carnegie Museum, v. 67, no. 1, p. 53-93.

Berman, D. S., Reisz, R. R., Martens, T., and Henrici, A. C., 2001, A new species of *Dimetrodon* (Synapsida: Sphenacodontidae) from the Lower Permian of Germany records first occurrence of genus outside of North America: Canadian Journal of Earth Sciences, v. 38, p. 803-812.

Blackburn, T. J., Olsen, P. E., Bowring, S. A., McLean, N. M., Kent, D. V., Puffer, J., McHone, G., Rasbury, E. T., and Et-Touhami, M., 2013, Zircon U-Pb geochronology links the end-Triassic extinction with the Central Atlantic Magmatic Province: Science, v. 340, no. 6135, p. 941-945.

Bolt, J. R., 1980, New tetrapods with bicuspid teeth from the Fort Sill locality (Lower Permian, Oklahoma): Neues Jahrbuch für Geologie und Paläontologie, Monatschefte, v. 1980, no. 8, p. 449-459.

Boy, J. A., and Martens, T., 1991, Ein neues captorhinomorphes reptil aus dem Thüringischen Rotliegend (Unter-Perm; Ost-Deutschland): Paläontologische Zeitschrift, v. 65, no. 3/4, p. 363-389.

Burgess, S. D., Bowring, S., and Shen, S.-z., 2014, High-precision timeline for Earth's most severe extinction: Proceedings of the National Academy of Sciences, v. 111, no. 9, p. 3316-3321.

Calder, J. H., Rygel, M. C., Ryan, R. J., Falcon-Lang, H. J., and Hebert, B. L., 2005, Stratigraphy and sedimentology of early Pennsylvanian red beds at Lower Cove, Nova Scotia, Canada: the Little River Formation with redefinition of the Joggins Formation: Atlantic Geology, v. 41, p. 143-167.

Carroll, R. L., 1964, The earliest reptiles: Zoological Journal of the Linnean Society, v. 45, no. 304, p. 61-83.

Carroll, R. L., 1969, A middle Pennsylvanian captorhinomorph, and the interrelationships of primitive reptiles: Journal of Paleontology, v. 43, no. 1, p. 151-170.

Carroll, R. L., and Baird, D., 1972, Carboniferous stem-reptiles of the family Romeriidae: Bulletin of the Museum of Comparative Zoology, v. 143, no. 5, p. 321-364.

Cisneros, J. C., 2008, Phylogenetic relationships of procolophonid parareptiles with remarks on their geological record: Journal of Systematic Palaeontology, v. 6, no. 3, p. 345-366.

Cisneros, J. C., Rubidge, B. S., Mason, R., and Dube, C., 2008, Analysis of millerettid parareptile relationships in the light of new material of *Broomia perplexa* Watson, 1914, from the Permian of South Africa: Journal of Systematic Palaeontology, v. 6, no. 4, p. 453-462.

Clark, J., and Carroll, R. L., 1973, Romeriid reptiles from the Lower Permian: Bulletin of the Museum of Comparative Zoology, v. 144, no. 5, p. 353-407.

Day, M. O., Ramezani, J., Bowring, S. A., Sadler, P. M., Erwin, D. H., Abdala, F., and Rubidge, B. S., 2015, When and how did the terrestrial mid-Permian mass extinction occur? Evidence from the tetrapod record of the Karoo Basin, South Africa: Proceedings of the Royal Society of London, Biological Sciences, v. 282, p. 20150834, 20150831-20150838.

Dodick, J. T., and Modesto, S. P., 1995, The cranial anatomy of the captorhinid reptile *Labidosaurikos meachami* from the Lower Permian of Oklahoma: Palaeontology, v. 38, no. 3, p. 687-711.

Dolby, G., 1991, The palynology of the western Cumberland Basin, Nova Scotia: Nova Scotia Department of Mines & Energy Open-File Report, v. 91-006, p. 1-51.

Dolby, G., 2003, Palynological analysis of ten outcrop & corehole samples from Nova Scotia: Nova Scotia Department of Natural Resources Open-File Report, v. ME 2003-005, p. 1-7.

Fox, R. C., and Bowman, M. C., 1966, Osteology and relationships of *Captorhinus aguti* (Cope) (Reptilia: Captorhinomorpha): University of Kansas Paleontological Contributions, v. 11, p. 1-79.

Gibling, M. R., and Bird, D. J., 1994, Late Carboniferous cyclothems and alluvial paleovalleys in the Sydney Basin, Nova Scotia: Geological Society of America Bulletin, v. 106, p. 105-117.

Gradstein, F. M., Ogg, J. G., Schmitz, M. D., and Ogg, G. M., 2012, The Geologic Time Scale 2012: Oxford, Elsevier, p. 1144.

Heaton, M. J., 1979, Cranial anatomy of primitive captorhinid reptiles from the Late Pennsylvanian and Early Permian Oklahoma and Texas: Oklahoma Geological Survey Bulletin, v. 127, p. 1-84.

Heckel, P. H., 2013, Pennsylvanian stratigraphy of northern Midcontinent Shelf and biostratigraphic correlation of cyclothems: Stratigraphy, v. 10, no. 1-2, p. 3-39.

Heckel, P. H., Barrick, J. E., and Rosscoe, S. J., 2011, Conodont-based correlation of marine units in lower Conemaugh Group (Late Pennsylvanian) in northern Appalachian Basin: Stratigraphy, v. 8, no. 4, p. 253-269.

Hentz, T. F., 1989, Permo-Carboniferous lithostratigraphy of the vertebrate-bearing Bowie and Wichita groups, north-central Texas, in Hook, R. W., ed., Permo-Carboniferous Vertebrate Paleontology, Lithostratigraphy, and Depositional Environments of North-Central Texas, Field Trip Guidebook 2: Austin, Society of Vertebrate Paleontology, p. 1-21.

Hook, R. W., 1989, Stratigraphic distribution of tetrapods in the Bowie and Wichita groups, Permo-Carboniferous of north-central Texas, in Hook, R. W., ed., Permo-Carboniferous Vertebrate Paleontology, Lithostratigraphy, and Depositional Environments of North-Central Texas, Field Trip Guidebook 2: Austin, Society of Vertebrate Paleontology, p. 47-53.

Kalibová, M., 1989, Palynology of the Nýřany Member (Westphalian D) in the Mšeno Basin: Sborník Geologických Věd, Paleontologie, v. 30, p. 85-121.

Kalibová-Kaiserová, M., 1967, Erforschung der megasporen von den Nýřany flözen (Westfal D) des Böhmischen Karbons: Review of Palaeobotany and Palynology, v. 1, no. 1-4, p. 201-210.

Kissel, R., 2010, Morphology, phylogeny, and evolution of Diadectidae (Cotylosauria: Diadectomorpha) [Ph.D. dissertation Ph.D.]: University of Toronto, 185 p.

Kissel, R. A., and Reisz, R. R., 2004, Synapsid fauna of the Upper Pennsylvanian Rock Lake Shale near Garnett, Kansas and the diversity pattern of early amniotes, in Arratia, G., Wilson, M. V. H., and Cloutier, R., eds., Recent Advances in the Origin and Early Radiation of Vertebrates: München, Verlag Dr. Friedrich Pfeil, p. 409-428.

Klembara, J., 2011, The cranial anatomy, ontogeny, and relationships of *Karpinskiosaurus secundus* (Amalitzky) (Seymouriamorpha, Karpinskiosauridae) from the Upper Permian of European Russia: Zoological Journal of the Linnean Society, v. 161, p. 184-212.

Klembara, J., and Ruta, M., 2004, The seymouriamorph tetrapod *Utegenia shpinari* from the ?Upper Carboniferous-Lower Permian of Kazakhstan. Part I: cranial anatomy and ontogeny: Transactions of the Royal Society of Edinburgh: Earth Sciences, v. 94, p. 45-74.

Lucas, S. G., 2013, Vertebrate biostratigraphy and biochronology of the upper Paleozoic Dunkard Group, Pennsylvania-West Virginia-Ohio, USA: International Journal of Coal Geology, v. 119, p. 79-87.

Lucas, S. G., Harris, S. K., Spielmann, J. A., Berman, D. S., Henrici, A. C., Heckert, A. B., Zeigler, K. E., and Rinehart, L. F., 2005a, Early Permian vertebrate biostratigraphy at Arroyo del Agua, Rio Arriba County, New Mexico: New Mexico Museum of Natural History & Science Bulletin, v. 31, p. 163-169.

Lucas, S. G., Harris, S. K., Spielmann, J. A., Berman, D. S., Henrici, A. C., Heckert, A. B., Zeigler, K. E., and Rinehart, L. F., 2005b, Early Permian vertebrate assemblage and its biostratigraphic significance, Arroyo del Agua, Rio Arriba County, New Mexico: New Mexico Geological Society Guidebook, v. 56, p. 228-296.

Maddin, H. C., Sidor, C. A., and Reisz, R. R., 2008, Cranial anatomy of *Ennatosaurus tecton* (Synapsida: Caseidae) form the Middle Permian of Russia and the evolutionary relationships of Caseidae: Journal of Vertebrate Paleontology, v. 28, no. 1, p. 160-180.

Merrill, G. K., 1972, Taxonomy, phylogeny, and biostratigraphy of *Neognathodus* in Appalachian Pennsylvanian rocks: Journal of Paleontology, v. 46, no. 6, p. 817-829.

Michel, L. A., Tabor, N. J., Montañez, I. P., Schmitz, M. D., and Davydov, V. I., 2015, Chronostratigraphy and paleoclimatology of the Lodève Basin, France: evidence for a pan-tropical aridification event across the Carboniferous-Permian boundary: Palaeogeography, Palaeoclimatology, Palaeoecology, v. 430, p. 118-131.

Modesto, S. P., 1998, New information on the skull of the Early Permian reptile Captorhinus aguti: PaleoBios, v. 18, no. 2&3, p. 21-35.

Modesto, S. P., 1999, Observations on the structure of the early Permian reptile *Stereosternum tumidum* Cope: Palaeontologia Africana, v. 35, p. 7-19.

Modesto, S. P., 2006, The cranial skeleton of the Early Permian aquatic reptile *Mesosaurus tenuidens*: implications for relationships and palaeobiology: Zoological Journal of the Linnean Society, v. 146, p. 345-368.

Modesto, S. P., 2010, The postcranial skeleton of the aquatic parareptile *Mesosaurus tenuidens* from the Gondwanan Permian: Journal of Vertebrate Paleontology, v. 30, no. 5, p. 1378-1395.

Modesto, S., and Smith, R. M. H., 2001, A new Late Permian captorhinid reptile: a first record from the South African Karoo: Journal of Vertebrate Paleontology, v. 21, no. 3, p. 405-409.

Modesto, S. P., Scott, D. M., Berman, D. S., Johannes, M., and Reisz, R. R., 2007, The skull and the palaeoecological significance of *Labidosaurus hamatus*, a captorhinid reptile from the Lower Permian of Texas: Zoological Journal of the Linnean Society, v. 149, p. 237-262.

Müller, J., and Reisz, R. R., 2005, An early captorhinid reptile (Amniota, Eureptilia) from the Upper Carboniferous of Hamilton, Kansas: Journal of Vertebrate Paleontology, v. 25, no. 3, p. 561-568.

Müller, J., and Reisz, R. R., 2006, The phylogeny of early eureptiles: comparing parsimony and Bayesian approaches in the investigation of a basal fossil clade: Systematic Biology, v. 55, no. 3, p. 503-511.

Müller, J., Berman, D. S., Henrici, A. C., Martens, T., and Sumida, S. S., 2006, The basal reptile *Thuringothyris mahlendorffae* (Amniota: Eureptilia) from the Lower Permian of Germany: Journal of Paleontology, v. 80, no. 4, p. 726-739.

Olson, E. C., 1958, Fauna of the Vale and Choza: 14. Summary, review, and integration of the geology and the faunas: Fieldiana Geology, v. 10, no. 32, p. 397-448.

Olson, E. C., 1967, Early Permian vertebrates of Oklahoma: Oklahoma Geological Survey Circular, v. 74, p. 1-111.

Olson, E. C., 1970, New and little known genera and species of vertebrates from the Lower Permian of Oklahoma: Fieldiana Geology, v. 18, no. 3, p. 359-443.

Peabody, F. E., 1952, *Petrolacosaurus kansensis* Lane, a Pennsylvanian reptile from Kansas: University of Kansas Paleontological Contributions, v. 1, p. 1-41.

Piñeiro, G., Ferigolo, J., Ramos, A., and Laurin, M., 2012, Cranial morphology of the Early Permian mesosaurid *Mesosaurus tenuidens* and the evolution of the lower temporal fenestration reassessed: Comptes Rendus Palevol, v. 11, p. 379-391.

Price, L. I., 1937, Two new cotylosaurs from the Permian of Texas: Proceedings of the New England Zoölogical Club, v. 16, p. 97-102.

Reisz, R. R., and Fröbisch, J., 2014, The oldest caseid synapsid from the Late Pennsylvanian of Kansas, and the evolution of herbivory in terrestrial vertebrates: PLoS One, v. 9, no. 4, e94518, 1-9.

Reisz, R. R., Berman, D. S., and Scott, D., 1984, The anatomy and relationships of the Lower Permian reptile *Araeoscelis*: Journal of Vertebrate Paleontology, v. 4, no. 1, p. 57-67.

Reisz, R. R., Maddin, H. C., Fröbisch, J., and Falconnet, J., 2011, A new large caseid (Synapsida, Caseasauria) from the Permian of Rodez (France), including a reappraisal of "*Casea*" *rutena* Sigogneau-Russell & Russell, 1974: Geodiversitas, v. 33, no. 2, p. 227-246.

Ritter, S. M., 1995, Upper Missourian-lower Wolfcampian (upper Kasimovian-lower Asselian) conodont biostratigraphy of the midcontinent, U.S.A.: Journal of Paleontology, v. 69, no. 6, p. 1139-1154.

Romer, A. S., 1928, Vertebrate faunal horizons in the Texas Permo-Carboniferous red beds: University of Texas Bulletin, v. 2801, p. 67-108.

Rubidge, B. S., 2005, Re-uniting lost continents - fossil reptiles from the ancient Karoo and their wanderlust: South African Journal of Geology, v. 108, p. 135-172.

Rubidge, B. S., Erwin, D. H., Ramezani, J., Bowring, S. A., and de Klerk, W. J., 2013, High-precision temporal calibration of Late Permian vertebrate biostratigraphy: U-Pb zircon constraints from the Karoo Supergroup, South Africa: Geology, v. 41, no. 3, p. 363-366.

Sawin, R. S., Franseen, E. K., West, R. R., Ludvigson, G. A., and Watney, W. L., 2008, Clarification and changes in Permian stratigraphic nomenclature in Kansas: Kansas Geological Survey Bulletin, v. 254, no. 2, p. 1-4.

Schneider, J. W., and Werneburg, R., 2006, Insect biostratigraphy of the Euramerican continental Late Pennsylvanian and Early Permian: Geological Society of London Special Publication, v. 265, p. 325-336.

Schneider, J. W., Lucas, S. G., and Barrick, J. E., 2013, The Early Permian age of the Dunkard Group, Appalachian Basin, U.S.A., based on spiloblattinid insect biostratigraphy: International Journal of Coal Geology, v. 119, p. 88-92.

Sturgeon, M. T., and Youngquist, W., 1949, Allegheny conodonts from eastern Ohio: Journal of Paleontology, v. 23, no. 4, p. 380-386.

Sumida, S. S., 1987, Two different vertebral forms in the axial column of *Labidosaurus* (Captorhinomorpha: Captorhinidae): Journal of Paleontology, v. 61, no. 1, p. 155-167.

Sumida, S. S., 1989, The appendicular skeleton of the Early Permian genus *Labidosaurus* (Reptilia, Captorhinomorpha, Captorhinidae) and the hind limb musculature of captorhinid reptiles: Journal of Vertebrate Paleontology, v. 9, no. 3, p. 295-313.

Sumida, S. S., Berman, D. S., and Martens, T., 1996, Biostratigraphic correlations between the Lower Permian of North America and central Europe using the first record of an assemblage of terrestrial tetrapods from Germany: PaleoBios, v. 17, no. 2-4, p. 1-12.

Sumida, S. S., Berman, D. S., and Martens, T., 1998, A new trematopid amphibian from the Lower Permian of central Germany: Palaeontology, v. 41, no. 4, p. 605-629.

Vaughn, P. P., 1955, The Permian reptile *Araeoscelis* restudied: Bulletin of the Museum of Comparative Zoology, v. 113, no. 5, p. 305-467.

Vaughn, P. P., 1966, *Seymouria* from the Lower Permian of southeastern Utah, and possible sexual dimorphism in that genus: Journal of Paleontology, v. 40, no. 3, p. 603-612.

Wardlaw, B. R., 2005, Age assignment of the Pennsylvanian-Early Permian succession of north central Texas: Permophiles, v. 46, p. 21-22.

Williston, S. W., 1917, *Labidosaurus* Cope, a Lower Permian cotylosaur reptile from Texas: Journal of Geology, v. 25, no. 4, p. 309-321.

Woodhead, J., Reisz, R., Fox, D., Drysdale, R., Hellstrom, J., Maas, R., Cheng, H., and Edwards, R. L., 2010, Speleothem climate records from deep time? Exploring the potential with an example from the Permian: Geology, v. 38, no. 5, p. 455-458.

Zajíc, J., 2000, Vertebrate zonation of the non-marine Upper Carboniferous - Lower Permian basins of the Czech Republic: Courier Forschungsinstitut Senckenberg, v. 223, p. 563-575.

Zodrow, E. L., and Cleal, C. J., 1985, Phyto- and chronostratigraphical correlations between the late Pennsylvanian Morien Group (Sydney, Nova Scotia) and the Silesian Pennant Measures (south Wales): Canadian Journal of Earth Sciences, v. 22, p. 1465-1473.

Supplemental Table S2. Date ranges for Operational Taxonomic Units (OTUs) in Müller & Reisz (2006) (eureptiles and outgroups) used in a preliminary "practice". Dates derived from FossilWorks/the Paleobiology Database (PBDB).

Operational Taxonomic Units (OTUs)	Dates (Ma) max	min	Justification
Diadectomorpha	314.6	254	http://fossilworks.org/?a=taxonInfo&taxon_no=135806
Seymouriamorpha	280	270	Using *Seymouria* to stand-in for Semouriamorpha.
Caseidae	306.95	254	http://fossilworks.org/bridge.pl?a=taxonInfo&taxon_no=38913
Mesosauridae	290.1	279.5	http://fossilworks.org/bridge.pl?a=taxonInfo&taxon_no=37578
Millerettidae	265	252.3	http://fossilworks.org/bridge.pl?a=taxonInfo&taxon_no=37568
Procolophonidae	252.3	201.6	http://fossilworks.org/bridge.pl?a=taxonInfo&taxon_no=37522
Romeria_texana	296.4	268	http://fossilworks.org/bridge.pl?a=taxonInfo&taxon_no=134834&is_real_user=1
Protocaptorhinus	290.1	279.5	http://fossilworks.org/bridge.pl?a=taxonInfo&taxon_no=37505
Rhiodenticulatus	303.4	268	http://fossilworks.org/bridge.pl?a=taxonInfo&taxon_no=37503
Captorhinus_laticeps	290.1	279.5	http://fossilworks.org/bridge.pl?a=taxonInfo&taxon_no=134970
Captorhinus_aguti	279.5	272.5	http://fossilworks.org/bridge.pl?a=taxonInfo&taxon_no=90718
Labidosaurus	279.5	272.5	http://fossilworks.org/bridge.pl?a=taxonInfo&taxon_no=37501
Labidosaurikos	279.5	272.5	http://fossilworks.org/?a=taxonInfo&taxon_no=37500
Saurorictus	259	254	http://fossilworks.org/bridge.pl?a=taxonInfo&taxon_no=135442
Concordia	306.95	303.4	http://fossilworks.org/bridge.pl?a=taxonInfo&taxon_no=92484
Protorothyris	298.9	295	http://fossilworks.org/?a=taxonInfo&taxon_no=37494
Paleothyris	314.6	306.95	http://fossilworks.org/bridge.pl?a=taxonInfo&taxon_no=37493
Cephalerpeton	311.45	306.95	http://fossilworks.org/?a=taxonInfo&taxon_no=37490
Anthracodromeus	311.45	306.95	http://fossilworks.org/?a=taxonInfo&taxon_no=37487
Brouffia	311.45	306.9	http://fossilworks.org/?a=taxonInfo&taxon_no=37489
Coelostegus	311.45	306.9	http://fossilworks.org/?a=taxonInfo&taxon_no=37491
Hylonomus	318.1	314.6	http://fossilworks.org/?a=taxonInfo&taxon_no=37492
Thuringothyris	290.1	279.5	http://fossilworks.org/bridge.pl?a=taxonInfo&taxon_no=137222
Petrolacosaurus	305.9	303.4	http://fossilworks.org/bridge.pl?a=taxonInfo&taxon_no=37771
Araeoscelis	279.5	272.5	http://fossilworks.org/bridge.pl?a=taxonInfo&taxon_no=37773

Supplemental Table S3. Summary statistics on morphology data matrix from Müller & Reisz (2006). Calculated with *BEASTmasteR* functions.

OTU	Count of characters with data	Count of ambiguous	Count of "?"	Percentage complete
Diadectomorpha	125	6	1	94.70
Seymouriamorpha	128	0	4	96.97
Caseidae	131	0	1	99.24
Mesosauridae	127	0	5	96.21
Millerettidae	130	2	0	98.48
Procolophonidae	129	1	2	97.73
Romeria_texana	92	0	40	69.70
Protocaptorhinus	105	1	26	79.55
Rhiodenticulatus	113	1	18	85.61
Captorhinus_laticeps	130	0	2	98.48
Captorhinus_aguti	130	0	2	98.48
Labidosaurus	127	0	5	96.21
Labidosaurikos	100	0	32	75.76
Saurorictus	65	0	67	49.24
Concordia	92	0	40	69.70
Protorothyris	124	0	8	93.94
Paleothyris	126	0	6	95.45
Cephalerpeton	83	0	49	62.88
Anthracodromeus	67	0	65	50.76
Brouffia	108	0	24	81.82
Coelostegus	80	0	52	60.61
Hylonomus	105	0	27	79.55
Thuringothyris	118	0	14	89.39
Petrolacosaurus	129	0	3	97.73
Araeoscelis	129	0	3	97.73

Number of taxa	Total with data	Total ambiguous	Total "?"	Total complete (percent)	Total number of characters
25	2793	11	496	84.64	3300

Supplemental Table S4. Comparison of summary statistics from the five Beast2 runs using tip dates derived from the Paleobiology Database.

Run #	**6**	**7**	**8**	**9**	**10**
Data	Including autapomorphies		Excluding autapomorphies		
Model	M*k*	M*k*v	M*k*	M*k*v	M*k*-parsinf
Ln Posterior	-1393.4	-1364.6	-1158.7	-1149.1	-1137.7
ESS	1752	1582	1413	1801	1801
Root age	332.6 [330.3, 335.3]	332.5 [330.1 335.2]	332.7 [330.0, 335.2]	332.7 [330.0, 335.0]	332.6 [330.0, 335.0]
Birth	0.455 [0.040, 1.904]	0.334 [0.036, 1.165]	0.361 [0.040, 1.339]	0.443 [0.034, 1.812]	0.389 [0.037, 1.618]
Death	0.435 [3.24e-4, 1.91]	0.312 [1.32e-4, 1.161]	0.340 [7.54e-5, 1.370]	0.422 [2.97e-4, 1.803]	0.367 [1.14e-4, 1.613]
Sampling	0.023 [4.74e-4, 0.059]	0.024 [5.26e-4, 0.057]	0.024 [5.56e-4, 0.059]	0.023 [7.02e-4, 0.058]	0.024 [5.71e-4, 0.057]
Clock rate mean	0.095 [0.015, 0.230]	0.059 [0.008, 0.119]	0.902 [0.025, 4.535]	0.613 [0.015, 2.831]	0.240 [0.012, 0.811]
Clock rate SD	1.794 [1.199, 2.517]	1.769 [1.178, 2.511]	2.474 [1.567, 3.536]	2.403 [1.439, 3.395]	2.098 [1.281, 3.087]

Supplemental Table S5. Number of patterns that are unobservable in the Mk_{parsinf} model, which excludes parsimony-uninformative characters (invariant characters and autapomorphic characters). The ascertainment-bias correction requires that the likelihood be calculated for each of these patterns, which obviously becomes problematic when there are millions of such patterns. The calculations here assume unordered characters. This table duplicates Table 2, but is included in Supplemental Material for comparison. For ordered characters, see Supplemental Table S8.

		# states: 2	3	4	5	6
**# of taxa**	**4**	10	63	292	1045	3006
	**5**	12	93	544	2505	9276
	**10**	22	333	4084	42505	381546
	**20**	42	1263	32164	730005	15085086
	**50**	102	7653	500404	30062505	1698527706
	**100**	202	30303	4000804	490250005	57089105406
	**200**	402	120603	32001604	7921000005	1.87E+12
	**500**	1002	751503	500004004	3.11E+11	1.86E+14
	**1000**	2002	3003003	4000008004	4.99E+12	5.97E+15

Supplemental Table S6. Total number of possible patterns, determined by (# of states)^(# of taxa). Inf=above numeric limit of R.

		# states: 2	3	4	5	6
# of taxa	**4**	16	81	256	625	1296
	**5**	32	243	1024	3125	7776
	**10**	1024	59049	1048576	9765625	60466176
	**20**	1048576	3.487E+09	1.10E+12	9.54E+13	3.66E+15
	**50**	1.13E+15	7.18E+23	1.27E+30	8.88E+34	8.08E+38
	**100**	1.27E+30	5.15E+47	1.61E+60	7.89E+69	6.53E+77
	**200**	1.61E+60	2.66E+95	2.58E+120	6.22E+139	4.27E+155
	**500**	3.27E+150	3.64E+238	1.07E+301	Inf	Inf
	**1000**	1.07E+301	Inf	Inf	Inf	Inf

Supplemental Table S7. Fraction of the total number of possible patterns that are unobservable under M$k_{parsinf}$ ascertainment bias correction. The total number of possible patterns is determined by (# of states)^(# of taxa). For ordered characters, see Supplemental Table S10.

		# states: 2	3	4	5	6
# of taxa	**4**	0.625	0.777778	1.140625	1.672	2.319444
	**5**	0.375	0.382716	0.531250	0.8016	1.192901
	**10**	0.021484	0.005639	0.003895	0.004353	0.006310
	**20**	0.000040	3.6E-07	2.9E-08	7.7E-09	4.1E-09
	**50**	9.1E-14	1.1E-20	3.9E-25	3.4E-28	2.1E-30
	**100**	1.6E-28	5.9E-44	2.5E-54	6.2E-62	8.7E-68
	**200**	2.5E-58	4.5E-91	1.2E-113	1.3E-130	4.4E-144
	**500**	3.1E-148	2.1E-233	4.7E-293	~0	~0
	**1000**	1.9E-298	~0	~0	~0	~0

Supplemental Table S8. Number of patterns that are unobservable in the M$k_{parsinf}$ model, assuming ordered characters. For unordered characters, see Supplemental Table S5.

# of taxa	# states: 2	3	4	5	6
**4**	10	27	52	85	126
**5**	12	33	64	105	156
**10**	22	63	124	205	306
**20**	42	123	244	405	606
**50**	102	303	604	1005	1506
**100**	202	603	1204	2005	3006
**200**	402	1203	2404	4005	6006
**500**	1002	3003	6004	10005	15006
**1000**	2002	6003	12004	20005	30006

Table S9. Total number of possible patterns, determined by (# of states)^(# of taxa). Inf=above numeric limit of R.

		# states: 2	3	4	5	6
# of taxa	**4**	16	81	256	625	1296
	**5**	32	243	1024	3125	7776
	**10**	1024	59049	1048576	9765625	60466176
	**20**	1048576	3.487E+09	1.10E+12	9.54E+13	3.66E+15
	**50**	1.13E+15	7.18E+23	1.27E+30	8.88E+34	8.08E+38
	**100**	1.27E+30	5.15E+47	1.61E+60	7.89E+69	6.53E+77
	**200**	1.61E+60	2.66E+95	2.58E+120	6.22E+139	4.27E+155
	**500**	3.27E+150	3.64E+238	1.07E+301	Inf	Inf
	**1000**	1.07E+301	Inf	Inf	Inf	Inf

Supplemental Table S10. Fraction of the total number of possible patterns that are unobservable under M$k_{parsinf}$ ascertainment bias correction, assuming ordered characters. For unordered characters, see Supplemental Table S7.

		# states: 2	3	4	5	6
**# of taxa**	**4**	0.625	0.333333	0.203125	0.136	0.097222
	**5**	0.375	0.135802	0.062500	0.0336	0.020062
	**10**	0.021484	0.001067	0.000118	0.000021	0.000005
	**20**	0.000040	3.5E-08	2.2E-10	4.2E-12	1.7E-13
	**50**	9.1E-14	4.2E-22	4.8E-28	1.1E-32	1.9E-36
	**100**	1.6E-28	1.2E-45	7.5E-58	2.5E-67	4.6E-75
	**200**	2.5E-58	4.5E-93	9.3E-118	6.4E-137	1.4E-152
	**500**	3.1E-148	8.3E-236	5.6E-298	~0	~0
	**1000**	1.9E-298	~0	~0	~0	~0

Table S11. Comparison of summary statistics from the six Beast2 runs on a simulated "strict clock" dataset.

Run name	**01_Mk_on_strict_clock_alldata**	**02_Mk_on_strict_clock_variable**	**03_Mk_on_strict_clock_informative**	**04_Mkv_on_strict_clock_variable**	**05_Mkv_on_strict_clock_informative**	**06_MkParsInf_on_strict_clock_informative**
Data	all data (1000 characters)	variable only	Excluding autapomorphies	variable only	Excluding autapomorphies	Excluding autapomorphies
Model	M*k*	M*k*	M*k*	M*k*v	M*k*v	M*k*-parsinf
Ln Posterior	-3601.329	-2746.9	-1499.5	-2514.559	-1429.994	-1349.494
ESS	1403	1801	1517	1650	300	1582
Root age	5.419 [5.072, 5.835]	5.391 [5.030, 5.784]	5.881 [5.188, 6.563]	5.428 [5.0522, 5.8196]	5.906 [5.2275, 6.7353]	5.711 [5.1406, 6.3636]
Birth	0.716 [0.431, 1.062]	0.712 [0.418, 1.050]	0.768 [0.450, 1.185]	0.712 [0.4007, 1.0251]	0.76 [0.4372, 1.1874]	0.692 [0.3916, 1.0146]
Death	0.186 [2.8E-6, 0.548]	0.176 [4.0E-5, 0.535]	0.218 [4.8E-5, 0.664]	0.181 [4.9184E-5, 0.5401]	0.209 [5.0351E-5, 0.6635]	0.174 [1.6085E-4, 0.538]
Sampling	0.549 [0.323, 0.792]	0.553 [0.323, 0.779]	0.577 [0.336, 0.828]	0.549 [0.3373, 0.8044]	0.568 [0.337, 0.8512]	0.545 [0.3258, 0.788]
Clock rate mean	0.011 [9.8E-3, 0.0132]	0.0279 [0.0235, 0.032]	0.040 [0.030, 0.051]	0.006538 [2.3798E-3, 0.01]	0.008267 [4.3272E-3, 0.0226]	0.007353 [2.4202E-3, 0.0125]
Clock rate SD	0.144 [1.3E-4, 0.310]	0.159 [1.6E-4, 0.335]	0.398 [0.108, 0.668]	0.144 [4.1579E-4, 0.3052]	0.381 [0.1302, 0.6833]	0.31 [4.6372E-4, 0.5827]

Table S12. Comparison of summary statistics from the six Beast2 runs on a simulated "non-clock" dataset.

Run name	**07_Mk_on_no_clock_all data**	**08_Mk_on_no_clock_variable**	**09_Mk_on_no_clock_informative**	**10_Mkv_on_no_clock_variable**	**11_Mkv_on_no_clock_informative**	**12_MkParsInf_on_no_clock_informative**
Data	all data (1000 characters)	variable only	variable only	Excluding autapomorphies	Excluding autapomorphies	Excluding autapomorphies
Model	M*k*	M*k*	M*k*v	M*k*	M*k*v	M*k*-parsinf
Ln Posterior	-3483.454	-1550.787	-1127.077	-1087.535	-742.043	-711.542
ESS	1094	1703	1505	1508	1579	1617
Root age	6.044 [5.0236, 7.125]	6.028 [5.0307, 7.1057]	5.968 [5.0309, 7.1324]	6.019 [5.0062, 7.0972]	5.982 [5.0273, 7.194]	6.01 [5.0381, 7.0406]
Birth	0.805 [0.4334, 1.2643]	0.833 [0.4054, 1.3088]	0.887 [0.4514, 1.3682]	0.843 [0.4443, 1.3479]	0.899 [0.5134, 1.4143]	0.856 [0.4424, 1.3434]
Death	0.23 [7.7108E-5, 0.7063]	0.253 [4.0782E-4, 0.7777]	0.274 [9.3399E-5, 0.8591]	0.259 [2.5074E-4, 0.8102]	0.279 [1.4534E-4, 0.836]	0.257 [1.0318E-4, 0.7776]
Sampling	0.596 [0.3309, 0.877]	0.606 [0.3286, 0.9007]	0.642 [0.35, 0.9612]	0.605 [0.3175, 0.8897]	0.638 [0.3652, 0.9527]	0.612 [0.3231, 0.891]
Clock rate mean	0.0294 [0.0112, 0.0587]	0.02192 [9.1469E-3, 0.0388]	0.02674 [7.432E-3, 0.0517]	0.01484 [3.2239E-3, 0.0308]	0.03211 [5.1528E-3, 0.0756]	0.01501 [2.6756E-3, 0.0327]
Clock rate SD	1.425 [0.9995, 1.9628]	1.138 [0.6843, 1.5963]	1.377 [0.8141, 2.0006]	1.143 [0.682, 1.6458]	1.379 [0.8731, 2.0378]	1.154 [0.6475, 1.6884]
